# Multi-scale imaging and analysis identify pan-embryo cell dynamics of germlayer formation in zebrafish

**DOI:** 10.1038/s41467-019-13625-0

**Published:** 2019-12-17

**Authors:** Gopi Shah, Konstantin Thierbach, Benjamin Schmid, Johannes Waschke, Anna Reade, Mario Hlawitschka, Ingo Roeder, Nico Scherf, Jan Huisken

**Affiliations:** 1 Max Planck Institute of Molecular Cell Biology and Genetics, Pfotenhauerstr. 108, 01307 Dresden, Germany; 2 European Molecular Biology Laboratory, Carrer del Dr. Aiguader, 88, 08003 Barcelona, Spain; 3 Institute for Medical Informatics and Biometry, Carl Gustav Carus Faculty of Medicine, TU Dresden, 01307 Dresden, Germany; 4 Max Planck Institute for Human Cognitive and Brain Sciences, Stephanstr. 1a, 04103 Leipzig, Germany; 5 Optical Imaging Centre Erlangen, Friedrich-Alexander-University of Erlangen-Nuremberg, 91054 Erlangen, Germany; 6 Faculty of Computer Science and Media, Leipzig University of Applied Sciences, 04277 Leipzig, Germany; 7 Cardiovascular Research Institute, University of California, San Francisco, CA 94158-9001 USA; 8 Department of Biochemistry and Biophysics, University of California, San Francisco, CA 94158-2517 USA; 9 Morgridge Institute for Research, 330 N Orchard St, Madison, WI 53715 USA

**Keywords:** Gastrulation, Light-sheet microscopy

## Abstract

The coordination of cell movements across spatio-temporal scales ensures precise positioning of organs during vertebrate gastrulation. Mechanisms governing such morphogenetic movements have been studied only within a local region, a single germlayer or in whole embryos without cell identity. Scale-bridging imaging and automated analysis of cell dynamics are needed for a deeper understanding of tissue formation during gastrulation. Here, we report pan-embryo analyses of formation and dynamics of all three germlayers simultaneously within a developing zebrafish embryo. We show that a distinct distribution of cells in each germlayer is established during early gastrulation via cell movement characteristics that are predominantly determined by their position in the embryo. The differences in initial germlayer distributions are subsequently amplified by a global movement, which organizes the organ precursors along the embryonic body axis, giving rise to the blueprint of organ formation. The tools and data are available as a resource for the community.

## Abstract (web summary)

The precise cell dynamics of early development have not yet been visualized. Here, the authors use custom 4-lens light sheet microscopy to image and analyze the dynamics of all three fluorescently labeled germlayers, yielding a comprehensive, pan-embryo description of early zebrafish gastrulation.

## Introduction

In any complex system, it is predominantly the interactions of its parts, within and across scales, that lead to the emergence of structure and function^1^. The development of an organism from a single cell is a prime example of such a complex dynamic system, wherein an embryo forms through interactions of molecules to specify cells, cells forming tissues and tissues morphing into organs. In turn, morphological changes at the tissue level feedback to individual cell behaviors and a cell’s state influences intracellular molecular dynamics. The coordination of events across these scales ensures precise formation and positioning of organs during development. To understand how information flows across scales and progenitor cells organize into functional organs in a developing embryo, it is crucial to have a pan-embryo view of cell and gene expression dynamics with high enough spatial and temporal resolution to follow the course of individual cells, as well as entire tissues simultaneously.

Here we focus on zebrafish gastrulation, which offers a unique vertebrate system to study embryo-wide cell dynamics in vivo. Morphogenetic movements (epiboly, involution, convergence, and extension) during gastrulation dynamically organize a mass of undifferentiated cells into three distinct germlayers, ectoderm, mesoderm and endoderm, which give rise to specific organs, laying out the primary body plan^2^ (Supplementary Fig. 1). There is strong evidence that mechanical cell properties (e.g., differential adhesion and surface tension)^3–5^ and dynamic behaviors (random vs. directed cell movement, division, and intercalation)^6–10^ drive the formation of individual germlayers. However, mechanisms governing such movements^2^ have so far only been studied within a local region^11,12^, a single germlayer^6^ or in whole embryos without cell identity^13,14^. Thus, a holistic perspective is still missing.

Light sheet microscopy has opened up the possibility to image living organisms in toto over time at high resolution with its low photo-toxicity, fast acquisition and deep optical penetration^15^. However, visualizing the interplay of all germlayers simultaneously across the entire intact developing embryo requires multi-view, multi-color, three-dimensional time-lapse imaging, and analysis that goes beyond conventional imaging. Moreover, since sufficiently high resolution is required at all relevant scales, in toto imaging inevitably creates enormous amounts of data that impose entirely novel challenges on data handling and downstream analysis. Custom microscope hardware and software is needed to streamline acquisition and real-time data processing to avoid accumulating superfluous data. Only home-built systems offer the flexibility to directly access image voxels to reject background data and limit the acquisition to relevant data on the fly. Further, we need computational tools to navigate, summarize, and analyze the data from individual tracks of tens of thousands of cells across scales, as well as to extract tissue and organ morphogenetic movements from the dynamics of individual cells. Hence, in order to visualize complex developmental dynamics, the goal should be to provide an overview, while still retaining the ability to zoom into details where necessary^16^.

In this work, we set out to map the multi-scale dynamics in zebrafish gastrulation. We combine genetic tools to reveal individual cells and their tissue identity, fast long-term imaging with single cell resolution across the entire embryo and a multi-scale computational analysis framework based on single cell tracking that allows us to visualize different aspects of zebrafish gastrulation by integrating the necessary information across scales. We further provide an interactive, web-based visualization of the data as a resource for exploratory data analysis to the community.

## Results

### Pan-embryo cell-level visualization of germlayer dynamics

As a first step towards understanding the formation of germlayers at a global scale, we established a method to visualize cellular dynamics and germlayer identity with single cell resolution across the entire embryo. We co-expressed three fluorescent reporters in single zebrafish embryos: *Tg(sox17:H2B-tBFP)* (endodermal marker; nuclear), *Tg(mezzo:eGFP)*^17^ (pan-mesendodermal marker; cytoplasmic), and *Tg(h2afva:h2afva-mCherry)*^18^ (ubiquitous marker; nuclear). We chose this combination of markers to facilitate a subsequent, computational separation of the three germlayers from the acquired images. Depending on the presence of specific combinations of reporters we could distinguish: epiblast (used here to denote all blastomers at onset of gastrulation, and prospective ectoderm plus EVL towards end of gastrulation) expressing only *histone*, mesendoderm expressing *histone* and *mezzo*, while endoderm expressing all three reporters (Supplementary Fig. 2; Supplementary Movie 1). With such unique expression patterns for each germlayer the identity of each cell was found by simple subtraction of channel intensities. This resulted in nuclear expression of markers in all germlayers, providing near-identical signal in each germlayer for comparable cell segmentation efficiency and downstream analysis (Supplementary Fig. 2; Methods).

Visualization of germlayer formation requires fast and minimally invasive imaging with cellular resolution over hours. We built a custom 4-lens Selective Plane Illumination Microscopy (SPIM) setup^19^ capable of performing multi-view imaging of the triply labeled embryos with high acquisition speed (~30 s per time point), achieving cellular-resolution across the entire zebrafish embryo from 4–18 hpf. In order to limit the acquisition to relevant data only and thereby eliminate non-informative background in real-time, we acquired a 300 µm thick spherical shell around the embryo surface every 150 s (Supplementary Fig. 3). So only about 50% of the data needed to be transferred, stored and analyzed, while still faithfully capturing the 3D nature of germlayer formation. For embryos with non-spherical geometries there are also adaptive sampling schemes that could be used instead^20^.

**Fig. 1:**
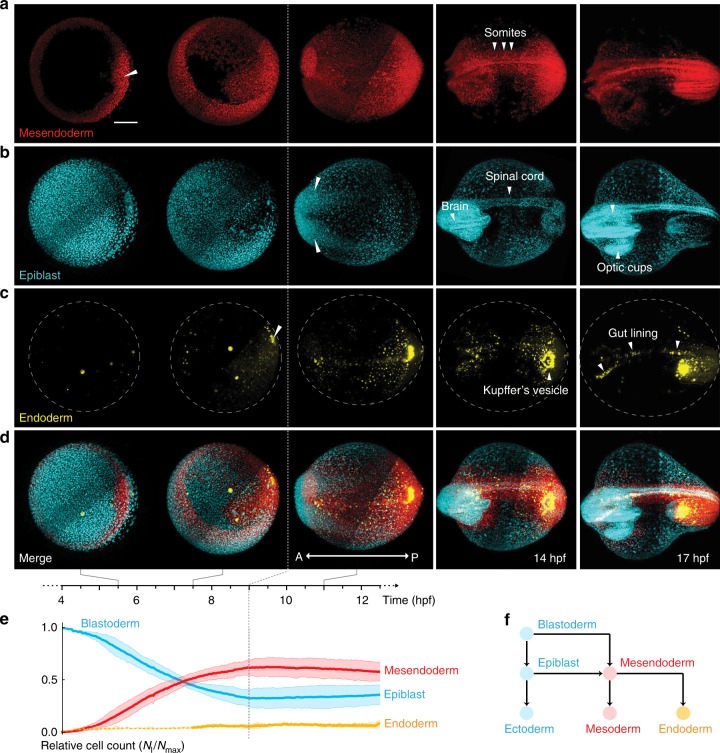
In toto imaging of germlayer specification and dynamics. **a**–**c** Formation and dynamics of mesendoderm (red), epiblast (cyan), and endoderm (yellow) cells spanning 4–17 hpf. *n* = 3 where all embryos resembled the images shown here. **a** Mesendoderm specification begins at the dorsal lip (white triangle), spreads around the germ ring and converges towards the dorsal midline, forming the somites and notochord (white triangles). **b** Epiblast cells converge towards the anterior (white triangles) leading to formation of brain, spinal cord and optic cups. **c** Endoderm specification begins at the dorsal shield (white triangle) with the DFCs, followed by rest of the endoderm, which forms the gut lining and Kupffer’s vesicle upon dorsal convergence. Yellow spots in the first two images is non-specific signal. **d** Merge of all three germlayers. Scale bar: 200 μm. A: anterior, P: posterior. **e** Mean relative cell numbers (*n* = 3) for epiblast (blue), mesendoderm (red) and endoderm (yellow). Bands correspond to 1.96 · standard error. Dashed part of the yellow line indicates the period before endoderm specification. **f** Schematic explaining germlayer specification showing blastoderm differentiating into mesendoderm and epiblast. Mesendoderm further differentiates into mesoderm and endoderm.

These data allowed us to explore the unfolding layer formation process at different levels of detail. Looking at the spatio-temporal dynamics of *mezzo* expression, one of the first nodal-activated transcription factors expressed in mesendoderm^21^, showed that differentiation of blastoderm into mesendoderm is a gradual process, beginning at 4.5 hpf at the dorsal lip and spreading laterally to cover the entire germ ring (Fig. 1a). The remaining blastoderm cells were used as an indirect readout of ectoderm, termed here as epiblast (this defines the total cell population that does not express mesendodermal markers; epiblast cells can still differentiate to form mesendoderm during development but correlate to ectoderm layer towards the end of gastrulation) (Fig. 1b, f). At 6.5 hpf, the dorsal forerunner cells (DFC) and the endoderm were formed as seen by the expression of *sox17*, one of the last transcription factors in the cascade for endoderm specification (Fig. 1c, f). We detected these cells and quantified their relative abundance in each germlayer from 4–12 hpf, which provided a comprehensive, quantitative depiction of germlayer formation. Relative cell counts of the three germlayers reached a plateau by 9 hpf (Fig. 1e) and all germlayers engaged thereafter in convergence-extension movements. Interestingly, epiblast cells moved to the anterior of the embryo, while converging towards dorsal to form the brain, spinal cord and optic cups (Fig. 1b, d). Mesendoderm cells converged towards mid-dorsal, as well as anterior and posterior parts, with mesoderm giving rise to somites and notochord (Fig. 1a, d) and endoderm forming the gut lining along with the assembly of Kupffer’s vesicle (Fig. 1c, d). Imaging the embryo-wide expression patterns of *histone*, *mezzo*, and *sox17* has allowed us to visualize the dynamics of all germlayers simultaneously within the developing embryo (Supplementary Movies 1, 2).

### Single cell motion patterns during early gastrulation

To understand if there are distinct cell movement patterns in different germlayers during gastrulation, we segmented and tracked cells in each germlayer separately (Supplementary Movies 3, 4). However, faithfully tracking 10,000 s cells over 14 h pushes the limit of existing algorithms due to the high local cell densities. Classifying the cells into distinct germlayers allowed us to study germlayer-specific motion in parallel, reducing the local density of cells to be tracked for each germlayer and leading to higher fidelity in cell segmentation and tracking.

**Fig. 2:**
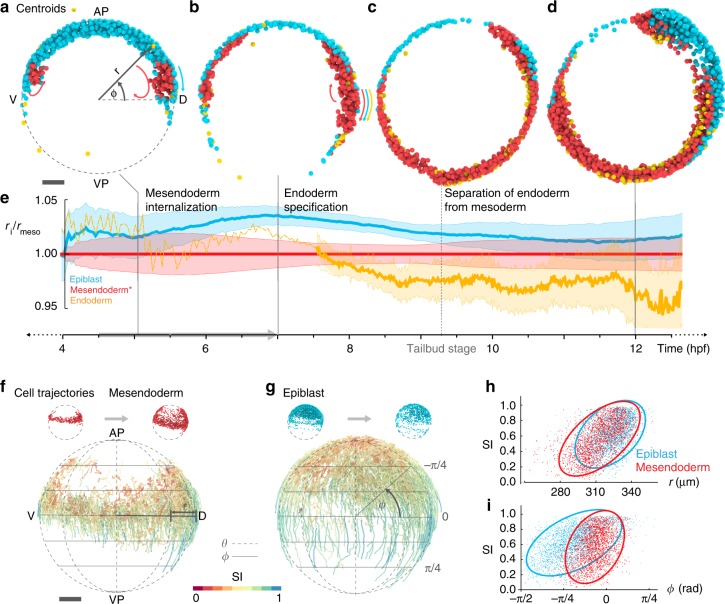
Position dependent organization of cell movement during early gastrulation. **a**–**d** Lateral views of the embryo at 4, 6.5, 9, and 11.5 hpf showing rendered object centroids located within a plane through the body axis (± 78 μm). Colors indicate the corresponding germlayer: epiblast (blue) mesendoderm (red) and endoderm (yellow). Colored arrows in (**a**) indicate epiboly of epiblast (blue) and internalization of mesendoderm (red), in **b** indicate epiboly movement of all three germlayers. AP: animal pole, VP: vegetal pole, D: dorsal, V: ventral. **e** Line plot showing mean radial position of all germlayers (normalized with respect to average radius of the mesendoderm) for a single embryo. Bands indicate mean + /− 0.3 standard deviation, the scaling was introduced to reduce overlap between germlayers and to visually highlight the thinning of layers. Dashed yellow line indicates the period before endoderm specification. **f** Cell trajectories for mesendodermal cells during early gastrulation (4.5–7 hpf; indicated by gray arrow in **e**) shown in lateral view. Color code indicates the straightness indices (SI) of trajectories. **g** Cell trajectories for epiblast cells, same views and color code as in **f**. **h** Scatterplot of SI vs. radial position r (computed at the midpoint) for each trajectory (4.5–7 hpf) of mesendoderm (red), epiblast (blue) and the respective 90% prediction ellipsoids. **i** Scatterplot of SI vs. position Φ along the longitude (computed at the midpoint) for each trajectory (4.5–7 hpf) of mesendoderm (red), epiblast (blue) and the respective 90% prediction ellipsoids.

Cell trajectories depicting the movement of individual cells within a germlayer and their spatial correlation throughout the process are shown in Supplementary Movies 5 and 6. During early gastrulation (4–7 hpf), a subpopulation of *mezzo* expressing cells involuted at 5.5 hpf forming a multi-layered mesendoderm. Subsequently, they moved towards the animal pole, sliding along the outer epiblast cells undergoing epiboly^11^ (Fig. 2a, b). All three germlayers continued their epiboly movement towards the vegetal pole to spread over the yolk (Fig. 2b). Though endoderm cells formed around 6.5 hpf, they retained their salt and pepper distribution with mesoderm cells as previously reported^8^, and separated from the mesoderm at the end of epiboly (Fig. 2b–d; Supplementary Fig. 4; Supplementary Movie 5). Through this complex execution of epiboly and internalization of cells, the desired radial stratification and thinning of layers was accomplished by ca. 9 hpf, as shown by the radial position of germlayers normalized to the mesendoderm position at each time point (Fig. 2e).

Several attempts have been made to find an order in this complex process, assuming germlayer specific cell behaviors: the endoderm has been shown to perform random walk after involution followed by directed dorsal-ward movement^6^, the ectoderm is thought to perform cell intercalation and collective cell movement^2,9,22^, whereas the mesoderm has been shown to exhibit a variety of behaviors along the dorso-ventral axis to pattern its motion^7,10,22,23^. To assess if the characteristics of cell movement (random vs. directed) are indeed germlayer specific, we measured how straight cells in each germlayer moved and correlated the straightness indices (SI) of cell trajectories to each of *r* (radius), *θ* (longitude), and *ϕ* (latitude) coordinates (methods). As tracking accuracy exponentially decays over trajectory length, we focused this analysis on short-scale cell tracks (10 frames ~20 min) where cell tracking was reliable. We found a striking pattern: both mesendoderm and epiblast cell tracks closer to the embryo surface (large r) exhibited a higher SI as compared to those at deeper locations (small *r*), revealing a radial organization (Fig. 2f–h). Likewise, epiblast cell tracks close to the animal pole (*θ* < −*π*/4) showed a lower SI than cells closer to the margin (*π*/4 < *ϕ* < −*π*/4), whereas no obvious pattern was observed along *ϕ* (Fig. 2h, i; Supplementary Fig. 5), indicating that the epiblast cell movement is also organized along the animal-vegetal axis of the embryo. This global perspective of single cell trajectories suggests that the straightness of cell movement strongly correlates with the cell’s position within the embryo (Fig. 2g, i), indicating a position-dependent rather than a germlayer specific organization of cell movement during early gastrulation.

**Fig. 3:**
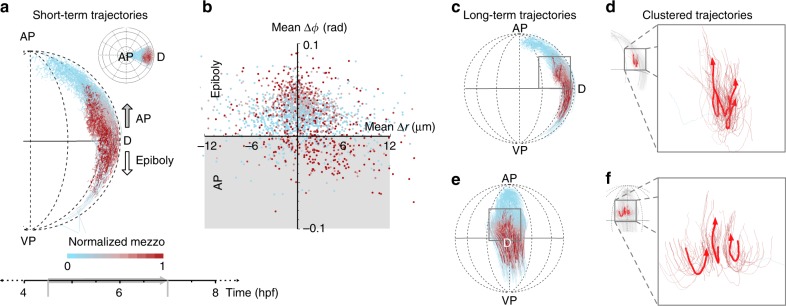
Cell movements during internalization. **a** Lateral view of the embryo showing movement of a subset of cells around shield region (4.5–7 hpf; selected subset of tracks shown in inset from animal pole). Arrows indicate direction of epiboly and internalization movement towards the animal pole. Color code shows normalized level of *mezzo* expression along each track. **b** Scatterplot showing the average change in radius and latitude for each track during 4.5–7 hpf time interval. Gray region indicates cells moving towards animal pole. Color code shows normalized *mezzo* expression. **c** Lateral view of long-term cell flows around shield region. Same region-of-interest as in **a**. Color code indicates normalized *mezzo* expression along track. **d** Lateral view of three clustered cell tracks undergoing internalization and movement towards animal pole. Thick lines show the representative cluster centroid trajectories. Magnified view of region indicated by box outline in **a**. **e** Dorsal view of long-term cell flows, same color code and region as in **a**. **f** Lateral view of the same clustered tracks and cluster centroids as in **d**.

Simultaneous with the epiboly movement, a population of mesendodermal cells undergo internalization and subsequently move towards the animal pole between 4.5–7 hpf (Fig. 2b). To understand if and how the migration patterns of those cells differ from the rest of the mesendoderm undergoing epiboly, we analyzed the internalization process in greater detail. During this period we also observed increasing *mezzo* expression of the mesendodermal cells, which might lead to a systematic bias when analyzing epiblast and mesendoderm separately, as cells that turn on *mezzo* expression are lost from the tracks of the initial epiblast population. Thus, to get an unbiased perspective of the internalization dynamics, we tracked all cells simultaneously during this period using solely the *histone* marker and extracted the *mezzo:GFP* signal separately for each tracked position (Methods). Since the internalization movement was most prominent at the shield, we focused on this region for the detailed analysis by considering only cell tracks with a longitude *ϕ* in the range [−*π*/8, *π*/8] (Fig. 3a). Also for the single cell tracks we observed a clear thickening of the inner tissue, as shown in the lateral view (Fig. 3a). The tracking data showed a subpopulation of mesendodermal cells undergoing an inward drift followed by a drift towards the animal pole, whereas the rest of the mesendoderm and epiblast followed epiboly motion (Fig. 3a, Supplementary Fig. 7, cf. Fig. 2b, Supplementary Movie 5). To understand how stereotypical and directed this internalization motion was, we looked into properties of the respective single-cell tracks. Plotting the average change in radius and latitude for each single track (Fig. 3b, Methods) clearly showed a predominantly mesendodermal subpopulation with negative radial and latitudinal shifts corresponding to a motion profile that points inwards and up towards animal pole. The resulting single cell dynamics are qualitatively depicted (Supplementary Fig. 6a–f) in temporal intervals of 30 mins. Quantifying the straightness of cell tracks in this region (Supplementary Fig. 6g–l) provided evidence for a non-directed mechanism as we found the same pattern of random motion inside of the multi-layered tissue and directed motion on the outer side (cf. Fig. 2h, i). To analyze the prototypical characteristics of cells undergoing internalization and animal pole movement dynamics, we used the statistical vector flow method^24^ to aggregate complete trajectories in the time window 4.5–7 hpf of small tissue regions from local, shorter cell tracks (Methods) resulting in the long term flows (Fig. 3c, e). We identified the respective subpopulation of internalizing mesendodermal cells by clustering the cell dynamics and selecting the cluster corresponding to internalization and animal pole movement for further analysis (Methods; Supplementary Fig. 8). To summarize the prototypical behavior of those cells, we further clustered the 3D trajectories of this subpopulation and computed the cluster centroids as representative trajectories (Fig. 3d, f). This information allowed us to schematically describe the internalization movement by integrating the data at different levels of granularity from single cell tracks and long-term flows (Supplementary Fig. 9): The internalizing fraction of mesendodermal cells drift inwards into the thickened tissue region above the yolk, and being blocked from epiboly move back towards the animal pole while undergoing convergence and radial outward motion (Fig. 3c–f, Supplementary Fig. 9k).

These findings suggest a strong influence of physical forces in this process: the internalization motion might not be an entirely active process where cells anticipate the movement autonomously as suggested^2^, but larger scale physical forces and geometric confinement, e.g., the inner wall of the tissue thickening at the yolk boundary, acting as a physical obstacle trapping a subpopulation of cells in the inner part at shield region, while the forces at the outer part of the embryo pull the outer cells over this region yielding the epiboly movement.

### From single cell motion to morphogenetic tissue movements

**Fig. 4:**
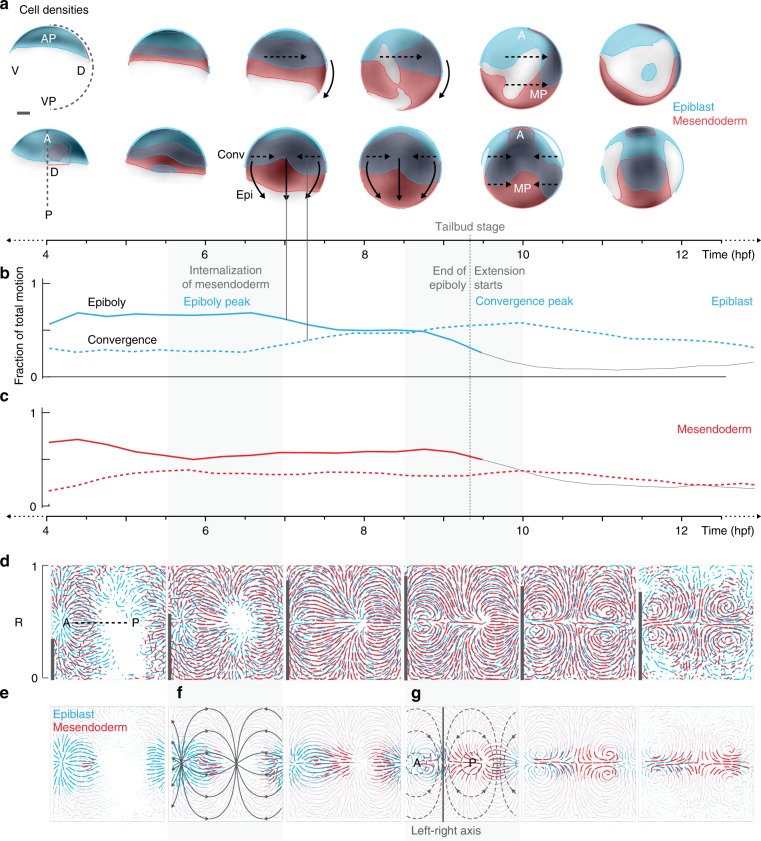
Distinct tissue flow patterns of germlayers during late gastrulation. **a** Cell densities shown as grayscale (white-low to black-high) on spherical representation of embryo. Regions of high densities (>0.33 normalized density) are color-coded, showing the extent of epiblast (blue) and mesendoderm (red) at various time points. Data shown in lateral (top) and dorsal (bottom) views. Arrows indicate expected direction of epiboly movement (solid) and convergence movement (dashed). AP: animal pole, VP: vegetal pole, MP: medio-posterior. **b**, **c** Proportions of epiboly (solid) and convergence (dashed) movement for epiblast (**b**) and mesendoderm (**c**) lines show mean across 3 embryos (*n* = 3). Gray dashed line indicates end of epiboly (tailbud stage). **d** General motion patterns of epiblast and mesendoderm shown as streamlines in Mercator projections, each interval covers ~1.5 h of development. Dorsal midline indicated by dashed line. Correlation of motion between germlayers is indicated as bars on the left edge of each streamline plot. **e** Tissue flow shown as density-weighted streamlines for epiblast and mesendoderm, thickness of streamlines indicates cell density at the respective site. Principal directions for epiboly (solid-gray) and convergence (dashed-gray) movement in Mercator projection are shown as overlay in **f** and **g**, respectively. **g** Border separating the ectodermal and mesendodermal flows is indicated by a solid line along the left-right axis.

Physical forces seem to play a prominent role in shaping the embryonic tissues, by distributing cells across the embryo depending on the radial positions of cells as seen for the separation of internalization and epiboly dynamics. To explore what shapes the layer-specific structures at the global level during development we next set out to systematically map the tissue-level flows across the embryo. We found a unique distribution of cells in each germlayer around 70% epiboly (7hpf) gastrulation, depicted by cell density rendering showing medio-posteriorly located mesendoderm and anteriorly located epiblast cells (Fig. 4a). This suggested that the different spatial distribution of cells within germlayers was important. However, it still remained to be understood if tissue dynamics are regulated individually for each germlayer or if they are governed by a global flow and the differences just arise from different initial spatial conditions. Exploring these tissue dynamics required a spatially coarse-grained analysis using the specific geometry of the specimen as a biological reference frame.

To investigate tissue motion, we analyzed the tracking results on a larger spatial scale by averaging the local direction of cell motion in spatial and temporal windows (Methods). We decomposed trajectories of every cell into an epiboly component (movement from animal to vegetal pole) and a convergence component (movement from ventral to dorsal side of the embryo) using the geometry of the specimen as a reference system^25^. In a spherical coordinate system of a suitably oriented embryo, convergence corresponds to motion along the parallels, whereas epiboly and extension correspond to directed motion along the meridians before and after completion of epiboly, respectively (cf. Supplementary Fig. 10). The radial movement of cells, which does not contribute directly to either of the movement components, was ignored in this analysis. Our results show that while the epiblast (ectoderm) undergoes a peak of epiboly followed by a peak of convergence in its movement, the mesendoderm has constant epiboly and convergence components throughout the process (Fig. 4b, c; Supplementary Fig. 11). Therefore, we hypothesize that the movements of epiblast (ectoderm) and mesendoderm cells are decoupled during late gastrulation as formerly postulated^2,26,27^ and that each germlayer possesses a characteristic motion pattern (as demonstrated for the endoderm in our previous study^19^).

### Global tissue flow patterns underlying germlayer dynamics

In order to visualize the spatial structure of germlayer specific motion patterns, we aggregated the information from the single cell tracks to the coarser tissue level by defining coarser spatial and temporal intervals. To this end, we merged cell-tracking data from multiple embryos, aggregated the directions of individual cell tracks in each spatial region over a certain time interval to obtain visualizations of cellular flows^28^ for each germlayer individually. They appeared strikingly similar, with a high correlation between the local flow directions of the mesendoderm and epiblast throughout the second phase of gastrulation (Fig. 4d). Hence, in contrast to our assumption that epiblast and mesendoderm cell movements are decoupled during late gastrulation, we found that a common global motion pattern drives the dynamics of all three germlayers simultaneously (Fig. 4d; Supplementary Fig. 12). Distinct patterns emerged when taking the spatial distribution of cells into account. To visualize this effect, the streamlines were scaled to reflect local cell densities of respective germlayers, indicating that the resultant organization of cells in each germlayer is dependent on the cell density distribution (Fig. 4e). Further, we found a separation of flows along the left-right axis between the epiblast and mesendoderm domains, which was strongest around 9 hpf (Fig. 4f, g). This boundary explains the prominent convergence of the epiblast towards the anterior and that of mesendoderm towards the medio-posterior region of the embryonic axis, as already apparent in Fig. 1 (Fig. 4e). Taken together, these results suggest that a common underlying flow drives large-scale formation of the embryonic body-plan. The localized initial distribution of mesendodermal precursors and the asymmetric flow shape the epiblast and the mesoderm layer into different patterns.

### Patterns of relative motion between germlayers

Although we found the large-scale tissue dynamics on the surface of the embryo to be very similar across the germ layers, this does not rule out local differences in cell motion that lead to a relative motion between layers, as we have shown for the internalization of mesendodermal cells at the shield region (Figs. 2f, 3). To get a global overview of these relative shifts between layers, we then mapped the global differences between the germlayer formation at the single cell and tissue level. To analyze these local differences at the single cell level, we first computed the correlation of cell movement (direction) between neighboring cells (Methods). Local cell motion is clearly correlated across layers in particular in the longitude (θ, motion along the dorso-ventral axis, e.g., convergence) and latitude (φ, motion along anterior-posterior axis, e.g., epiboly) and the temporal pattern showed peaks consistent with the peaks in epiboly and convergence motion at the tissue level (cf. Fig. 4b, c). However, the similarity of local movement direction was higher between cells of the same layer than between cells of different layers (Supplementary Fig. 13) indicating that there is indeed a residual, relative component in tissue formation between layers. To explicitly visualize the relative displacements between layers, we computed the direction of motion for each mesoderm cell across 10 min intervals and subtracted the average local movement of surrounding epiblast cells as proposed^29^ (Methods). The residual motion patterns across the embryonic surface showed a relative shift of the mesendodermal layer towards the anterior pole compared to epiblast movement (Supplementary Fig. 14a), consistent with the movement of mesendodermal cells towards the animal pole underneath the epiblast layer found at the single cell level (Fig. 3). The radial component of relative motion also highlights the tendency for mesodermal cells to move inwards as compared to epiblast cells at earlier stages from about 4.5 hpf to 7 hpf (Supplementary Figs. 14b, 9) corresponding to the tissue stratification during internalization of mesendoderm. Thus, while we’ve found a common large-scale tissue flow that is similar across layers, our analysis showed that relative motion between layers at the single cell level mostly results in local tissue rearrangements that organize the layers radially (and moves internalized mesendoderm towards the animal pole).

### Exploratory data analysis of long-term cellular pathways

**Fig. 5:**
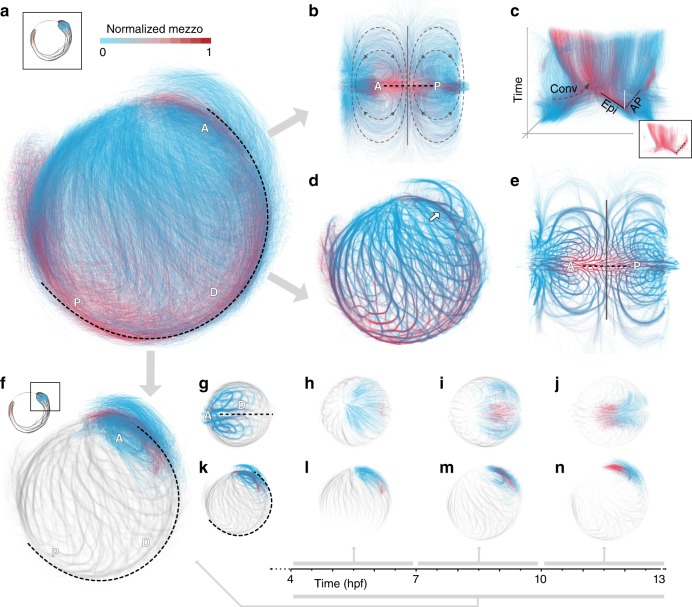
Exploratory data analysis of long-term pan embryo cell dynamics. **a** 3D rendering of long-term cell trajectories (A-anterior, P-posterior, dashed curve-dorsal midline). Color code shows normalized *mezzo* expression along each track. **b** 2D Mercator projection of cell tracks (A-anterior, P-posterior, dashed line-dorsal midline), same color code as in **a**. Principal directions for convergence (dashed-gray) movement and border separating the anterior and posterior flows (solid line along the left-right axis) are shown as overlays. **c** Space-time plots of long-term tracks over Mercator projection. *Z*-axis corresponds to time. Gray, dashed arrow illustrates the convergence movement (Conv). Dashed black line highlights movement of mesendodermal cells moving towards animal pole (AP). Solid black line indicates movement of mesendodermal cells undergoing epiboly (Epi). White line shows space-time line of a stationary object for reference. Inset shows subpopulation of cells with high normalized *mezzo* expression. Same color code as in **a**. **d** 3D rendering of bundled, long-term cell trajectories assembled from individual cell tracks (A-anterior, P-posterior, dashed curve-dorsal midline) highlighting structural details of cell migration patterns from 4 to 13 hpf. Color code shows normalized *mezzo* expression along track. Arrow indicates the internalized stream of mesendodermal cells towards animal pole. **e** 2D Mercator projection of bundled cell tracks reveals global structure of migration patterns across the developing embryo from 4 to 13 hpf (A-anterior, P-posterior, dashed line–dorsal midline). Border separating the anterior and posterior flows (solid line along the left-right axis) are shown as overlay. **f** Lateral view of selected anatomical region of interest. Long-term trajectories of cells within ROI are shown for 4–13 hpf time interval. Color code indicates normalized *mezzo* expression. Cell tracks outside of ROI shown as bundled tracks in gray. **g** Rotated, dorsal view of selected cell tracks shown in bundled state to highlight structure of cellular flows. **h**–**j** Backtracking of cells in ROI in time intervals of 90 min. Same color code as **f**. Tracks outside of region of interest are shown as gray bundles. **k**–**n** Lateral view of selected cell tracks, same visual coding as in **g**–**j**.

While we have analyzed local motion at the single cell level and long-term global tissue flows, it is equally important to visualize the actual paths taken by individual cells during gastrulation. In contrast to measurements intrinsically restricted at the tissue level (such as particle image velocimetry PIV), our single-cell data allowed us to visualize the tissue-level motion at single-cell resolution. However, simply plotting the 36,000 incomplete, individual cell tracks would not yield meaningful visualizations due to visual overlap of trajectories that would completely obscure the underlying patterns. To better reveal the long-term structure of single cell migration patterns, we used the tracks of all cells expressing *histone* and aggregated local cell tracks over the entire observation period to estimate long-range trajectories and computed the average *mezzo* reporter signal in a spatial window around the extracted cell centroids to map the cellular transition between germlayers (Methods). We further implemented an interactive software tool to explore and map the more than 10,000 resulting trajectories in real-time via a web browser interface. The interactive visualization provided an overview of the available tracking data and allowed the user to filter and map details on demand^16^ in a 3D view (Fig. 5a) or in 2D Mercator projections (Fig. 5b; Supplementary Fig. 15; Supplementary Movie 7, Methods). The 2D projection of the trajectories showed flow patterns that are consistent with the structure of the tissue flow fields (Fig. 4d, e). To visualize the temporal organization of large-scale morphogenetic flows, we showed time as an additional dimension over the 2D maps. These space-time plots summarized the morphogenetic dynamics during gastrulation (Fig. 5c) from a holistic perspective, e.g., the different time scales involved in movement of superficial cells (epiboly) and internalized mesendoderm towards animal pole are indicated by different slopes in the spacetime plot (Fig. 5c).

Still, one major limitation with tracking data is the visual overlap when plotting more than 10,000 trajectories at the same time. To reveal the underlying structure of the cell flows, we introduced edge-bundling to visually cluster similar cell paths and declutter the visualization (Methods). The bundled 3D cellular trajectories (Fig. 5d, e) highlight the radial germlayer stratification and the convergent movement of cells towards the dorsal part. It also clearly revealed the stream of internalized mesendoderm towards the animal pole region (arrow in Fig. 5d). The pan-embryo 2D mapping of the bundled paths (Fig. 5e) shows the morphogenetic patterns at the single cell level and highlights that convergent flows and the movement along the AP axis are gradual processes happening in parallel, consistent with the general flow from the tissue level analysis (cf. Fig. 4e). Importantly the long-term movement of mesendodermal and epiblast (ectoderm) cells are strikingly similar locally but differ globally due to the initial spatial asymmetry in the cell type distribution that yield spatially separated flows at the anterior and posterior regions (separating lines shown in Fig. 5b, e).

Tracking studies often aim to obtain the spatio-temporal distribution of organ progenitors, a subpopulation of cells at early time points that later give rise to specific organs. Our visualization tool allows to interactively filter for subpopulations of cells in anatomical regions of interest (ROI) and trace their dynamics across development, while still preserving the anatomical reference frame by showing the remaining trajectories (outside of the ROI) in a different visual encoding (Fig. 5f). As an illustrative example of tracing spatio-temporal lineage of an anatomical ROI, we selected a region corresponding to the head (brain and optic cups) (Fig. 5f) and visualized the bundled trajectories of all cells that end in this ROI (Fig. 5g, k), showing all remaining cell tracks in gray as reference. The traced bundles show that cells that contribute to the head region originate from the anterior part of the embryo (Fig. 5b, e and Fig. 4g). To reveal the temporal progression and spatial reorganization of the progenitors that contribute to the formation of the head region, we tracked the anatomical region across the entire observation period. Figure 5h–n shows the original, unbundled tracks with the respective *mezzo* readout in 3 h time intervals, highlighting the different spatial distribution and flows of internalized mesendoderm and converging epiblast cells. Thus, our multi-scale exploratory data analysis enables interactive, flexible reconstruction of the spatio-temporal origin of tissues at the cellular level.

## Discussion

In summary, our study shows that imaging and analysis of vertebrate development across scales is finally possible in vivo using a combination of genetic tools, fast light sheet imaging and multi-scale data analysis. We present a pan-embryo visualization of the differentiation of blastoderm into three germlayers and their dynamics at cellular resolution by using three distinct fluorophores to label the different tissues. The maturation times of the fluorophores used ranges between 10–60 min, which needs to be taken into account while inferring fates/lineages. However, for the length of time window we were interested in, fluorescent markers with long half-life provide an excellent readout of cell identity.

Through assessment of the process at the level of single cells, gene expression domains, entire germlayers and whole embryos simultaneously, we uncovered that during early gastrulation, spatially confined mesendoderm formation combined with the position dependent cell movement establishes a distinct initial distribution of cells in each germlayer. The differences in this initial distribution of cell types are amplified during late gastrulation, wherein a global movement organizes the organ precursors along the embryonic body axis, independent of their germlayer identity. In addition to the global movement, we uncovered local differences in cell motion between layers, which result in local rearrangements to obtain stratification of germlayers. This entire process gives rise to the blueprint of organ development in zebrafish. Gastrulation has been thought of as a complex process comprising multiple cell behaviors and movements driving different aspects of germlayer organization. Our analyses support the idea that gastrulation is in fact is a phenomenon of whole^30^, governed by global tissue flows^31^ and regulation of basic parameters such as initial cell positions, cell density and respective local mechanical interactions, as has been proposed earlier as a principle of development in other vertebrate organisms^5,32,33^. Such a mechanism relies on accurate differentiation of cells in space and time, as displacements in the initial mesendodermal population would get amplified and potentially lead to aberrant development. For example, displacement of endoderm precursor cells at the onset of gastrulation in cxcr4a morphants leads to mis-positioning of endoderm-derived organs, despite cell movements being unaffected^19^.

Being able to interactively visualize and explore the multi-scale developmental dynamics in wild-type zebrafish and to track cell cohorts from their inception to incorporation into specific organs will benefit developmental and disease-oriented studies^34^. Conventionally, such questions have been addressed through fate mapping experiments using sparse labeling that report spatial position of cells^35^. Here, we demonstrate that with the right combination of tools we can collect spatial and temporal information about large-scale cell movements that assemble specific organ precursors to form organs. This information complements the results from invasive, single-cell sequencing data taken at specific time points^36,37^. Furthermore, our interactive data visualization enables the user to explore, map, filter and analyze 10,000 s of single cell tracks in real time from any web browser. We hope that this will facilitate hypothesis generation and provide an explorable overview of gastrulation and other morphogenetic processes across scales.

We believe that our dataset together with the interactive, web-based visualization tool for exploratory analysis of the data (https://imb-dev.gitlab.io/cell-flow-navigator/) offer a valuable resource for the community to reconstruct the emergence and interplay of specific tissues and organs during early zebrafish development. Sharing of these long-term in toto data and the necessary analysis tools will be crucial for the community in its pursuit of understanding the fundamentals of development and we hope to have contributed in this regard.

## Methods

### Zebrafish

Zebrafish were handled in accordance with EU directive 2011/63/EU, as well as the German Animal Welfare Act. The transgenic zebrafish lines (*Tg(sox17:H2B-tBFP)*, *Tg(mezzo:eGFP)*^22^, and *Tg(h2afva:h2afva-mCherry)*^9^) were used for visualizing endodermal, mesendodermal and all cells, respectively, during gastrulation. For obtaining the triple transgenic embryos, a homozygous double transgenic line containing *Tg(mezzo:eGFP)* and *Tg(h2afva:h2afva-mCherry)* was generated and a female from this line was crossed with a homozygous *Tg(sox17:H2B-tBFP)* male fish. Embryos were collected after fertilization and incubated at 28.5 °C in E3 medium. To generate the *Tg(sox17:H2B-tBFP)* line, the transgene plasmid pTol2-sox17:H2B-tBFP was created by Gibson assembly^38^. The *sox17* promoter was PCR amplified from pSox17:eGFP^39^ and cloned into pminiTol2^40,41^ to generate pTol2-sox17. The H2B ORF and the tagBFP ORF were PCR amplified and cloned into pTol2-sox17 to generate pTol2-sox17:H2B-tBFP. This plasmid was used to generate the *Tg(sox17:H2B-tBFP)* stable zebrafish line using standard transgenesis protocols^40,41^.

### Sample preparation

Low-melting agarose (1.5%) solution with fluorescent beads (Merck Estapor fluorescent microspheres FXC-050 1:20,000 dilution) was prepared in E3 medium and maintained at 37 °C. At high stage, embryos with chorion were transferred into low-melting agarose and sucked into a cleaned FEP tube (inner diameter: 2.0 mm, wall thickness: 0.5 mm), using a micropipette (Eppendorf P200). 3 embryos were positioned on top of each other with minimum gap between them. The agarose was allowed to solidify at room temperature for 5 min and the FEP tube was mounted on the stage, dipping from the top into the sample chamber filled with E3 medium for time-lapse acquisition.

### SPIM hardware

A four-lens SPIM setup was used for imaging, consisting of four identical water-dipping Olympus UMPLFLN 10×/0.3 objectives, two for illumination and two for detection. The sample chamber was custom-made from acrylic with four openings sealed with rubber o-rings. Omicron SOLE-6 laser engine consisting of six different visible laser lines was mounted on the system and the laser beam through fiber was split into two for the two illumination arms. An optical chopper wheel (Thorlabs MC2000B, MC2F57Ba) was used to generate alternate illumination of the sample from two sides. The chopper wheel provided the start trigger for cameras and stages for image acquisition and its rotation speed was synchronized with the acquisition of the camera. Light sheets were generated with cylindrical lenses and projected with telescopes and the illumination objective lenses onto the common focal plane of both detection lenses. Light sheet thickness was calculated for half width of the field of view and their waists were centered in the respective halves of the FOV, resulting in a wider region with thinner light sheet. A continuously running resonant mirror (1 kHz, EOPC) was used in each illumination arm for even sample illumination (multidirectional SPIM). The average excitation power in the entire object plane was 8 mW per arm. The focal planes of the two detection objectives were imaged onto two Andor Zyla sCMOS cameras through emission filters (Chroma ET 460/50, ET BP525/50, Semrock BP593/46). Both cameras were precisely aligned using a focusable tube lens and an adjustable mirror to acquire images from the same focal plane, which were streamed to separate computers using camera controls implemented in Fiji. The sample was moved and rotated with three motorized linear stages (M-404.1PD/6PD, M-112.1DG) and one rotational stage (M-660.55, Physik Instrumente) using custom-written software in LabView specific to this instrument.

### Time-lapse acquisition

The setup consists of two illumination arms for dual-sided illumination of the sample and two detection arms to acquire two opposite views covering the entire embryo in ~10 s. The zebrafish embryo was moved along the detection axis through the alternating light sheets to acquire a z-stack of 402 planes. The two cameras were triggered simultaneously every 2 µm, acquiring one image stack for each combination of illumination side and camera, producing four image stacks per channel for each time point. The laser illuminates the sample only when the entire chip was exposed avoiding any artifacts due to the rolling shutter. These steps were repeated for sequential acquisition of the three channels, which took 30 s. Time points were acquired at an interval of 2 mins for a period of 14 h. To image multiple samples in parallel, the stage translated the tube along the *y*-axis to position the next embryo in front of the objective lenses. By repeating this, a time point is acquired for each sample and the stage moves back to bring the first embryo in the field of view. This entire process is repeated for time-lapse acquisition of multiple samples. In this study, three wildtype embryos were imaged simultaneously.

### Fitting a sphere to the transmission image

Before starting the time-lapse acquisition, a single image stack was acquired using transmission light. Due to the limited depth of field, different parts of the embryo were in focus in different planes of the transmission stack. A difference-of-Gaussian approach was used to filter efficiently for these in-focus regions:

1 $$\begin{array}{*{20}{c}} {I^\prime \left( {x,y,z} \right)\,=\,\left| {I\left( {x,y,z} \right) - G_\sigma \left( {x,y} \right)\,\times\,I\left( {x,y,z} \right)} \right|} \end{array}$$

where *I*(*x*,*y z*) denotes the intensity of the transmission image at pixel (x,y,z), *G*_*σ*_(*x*,*y*) is a 2D Gaussian kernel centered at (*x*,*y*) with a standard deviation of *σ* and * denotes plane-wise convolution. Throughout our experiments we used *σ* = 1.

Subsequently, a 3 × 3 median filter was applied to reduce noise. Two spheres were fitted to the resulting image stack by a custom algorithm motivated by the k-means algorithm. First, the image was roughly divided into foreground and background pixels by adjusting a global threshold. Foreground pixels usually covered the surface of the embryo, as well as the chorion. Two sphere models were then initialized, both centered at the image center and with estimated radii for the embryo and the chorion, 350 μm and 550 μm, respectively. An iterative, Expectation-Maximization-like algorithm was then executed, similar to the k-means algorithm: The expectation step assigned each foreground pixel to one of the sphere models, depending on its distance to the spheres’ surface. The maximization step updated both sphere models from the assigned pixels using a least-squares fit. Both steps were repeated until convergence. The sphere model with the smaller radius resembled the embryo surface.

### Real-time masked raw data acquisition

Since germlayer cells remain close to the surface of the zebrafish embryo throughout the experiment, only the pixels within a certain distance to the surface of the fitted sphere contain information that is of interest for further analysis. Since the position and size of the acquisition volume remain unchanged for all time points, we identify these pixels after sphere fitting and collect their indices, i.e., their linear position within the image stack, in a table. After acquiring each stack, instead of saving the data as ordinary image files, we save a concatenated list of intensity values of the identified pixels. The order of the pixels remains unchanged for consecutive stacks; to reassign intensity values later to pixel positions, we simply save the table with pixel indices once for all time points. This is done for each sample and for each view separately.

### Multi-view and time-lapse registration

The four views (image stacks) obtained from the combination of two illumination and two detection sides were fused using nonlinear blending to generate a fused image stack based on the transformations pre-calculated with the beads around the sample. This fusion is performed for each time point in the acquired time-lapse data.

As the embryonic axis forms towards the end of gastrulation, the embryo inside the chorion turns by 90°. To separate this global movement from local cell movements, 3D image stacks of consecutive time points were registered rigidly (Supplementary Movie 5). For this purpose, the images at each time point were smoothed using a Gaussian kernel with σ = 1. Local maxima above a certain threshold (th = 150 in our experiments), with neighbor maxima separated by at least 30 gray levels, identified the majority of cells centers. Due to the similarity between images of consecutive time points, our procedure detected a similar subset of cells that were subsequently used to estimate a rigid transformation using the Iterative Closest Point algorithm.

### Channel subtraction to separate germ layers

We recorded three transgenic lines (channels) at each time point, labeling the following layers: Channel 1 recording *Tg(sox17:H2B-tBFP)* expressed in Endoderm (nuclear expression), Channel 2 recording *Tg(mezzo:eGFP)* in Mesoderm + Endoderm (cytoplasmic expression) and Channel 3 recording Tg(h2afva:h2afva-mCherry) in Ectoderm + Mesoderm + Endoderm (nuclear expression). By subtracting channel 2 from channel 3 ubiquitously labeling nuclei of all three germ layers, a separate layer with epiblast (ectoderm) nuclei was obtained. Likewise, subtracting epiblast from the channel 3 gave mesendoderm layer with nuclear signal and further subtracting endoderm segregated mesoderm as well. Channel 1, labeling endoderm nuclei was used as it is for visualizing endoderm. This procedure yielded nuclear signals for all three germ layers. Each layer was then individually segmented and tracked for further analysis.

### Cell tracking

Cells were detected and tracked separately for each germ layer using the TGMM Software^42^. We set the backgroundThreshold manually for each layer and sample: (epiblast/mesoderm/endoderm): sample 1: 30/280/140, sample 2: 350/420/140, and sample 3: 250/150/120. The parameter anisotropyZ was set to 1.0, and thrCellDivisionWithTemporalWindow to 3.2 for all experiments. All other parameters were left at default values.

### *Histone* channel tracking and *mezzo:eGFP* signal readout

The nuclear Tg(h2afva:h2afva-mCherry) signal were detected and tracked for sample 1 (see *Cell tracking* above) using the TGMM Software^42^. We set the backgroundThreshold to 30, anisotropyZ to 1.0, and thrCellDivisionWithTemporalWindow to 3.2. All other parameters were left at default values.

We processed the TGMM tracks using the statistical vector flow (SVF) method from^24^ using default parameters. We then extracted the average *Tg(mezzo:eGFP)* signal in a spatial window of 3 × 3 × 3 voxels around the extracted cell positions at each time point. We normalized the *mezzo* signal readout to the range [0,1] using the minimum and maximum value of the *Tg(mezzo:eGFP)* across all available images.

### Spatial alignment of data sets

Raw tracking results were transformed into a standardized Cartesian coordinate system for each specimen: the origin of this coordinate system was moved to the center of the embryo by subtracting the centroid position of the best-fit sphere from each observed position. The radial position of each cell was then calculated as the length of its (centered) position vector ||(*x*,*y*,*z*)||. Remaining differences in orientation between datasets were corrected manually by aligning the data with respect to a reference embryo, using the start and end of the movie, as well as 75% epiboly stage as reference time points.

### Cell counts

Cell numbers *n*_*i*_ for each germ layer were estimated using the number of detected objects at frame i. Relative cell numbers were obtained by normalizing the per-layer cell counts with the total cell number of cells in all three germ layers at each frame i.

### Regularity of cell movement

To characterize how directed a cell’s movement is, we calculated the straightness index (SI)^43^ by normalizing the end-to-end distance by the actual path length of a trajectory:

2 $$\begin{array}{*{20}{c}} {SI\left( {{\boldsymbol{x}}_i} \right) = \frac{{\left| {\left| {{\boldsymbol{x}}_i\left( {n_i} \right) - {\boldsymbol{x}}_i\left( 1 \right)} \right|} \right|}}{{\mathop {\sum }\nolimits_{k = 1}^{N_i - 1} {\boldsymbol{x}}_i\left( {k \, + 1} \right) - {\boldsymbol{x}}_i\left( k \right)}},} \end{array}$$

With || || being the Euclidean norm, ***x***_*i*_(*k*), being the ith cell’s positions at time point k in Cartesian coordinates: [***x***_*i*_(*k*),***y***_*i*_(*k*),***z***_*i*_(*k*)], and *N*_*i*_ being the length of trajectory i.

### Statistical analysis of cell motion patterns

The influence of different intrinsic and extrinsic factors on straightness of cell motion was first assessed by scatter plots of cell position (midpoint of the track) vs. the respective SI of the cell’s track, together with the 90% prediction ellipses enclosing the region, where 90% of new observations would be located under the condition that the data is drawn from a bivariate normal distribution.

### Analysis of average change in radius and latitude

Cell tracks were selected in a temporal interval from 4.5–7 hpf. For each track the change in radius and latitude was computed by first computing the difference between the last and the first position (in spherical coordinates) for non-overlapping pieces of 10 min duration and then averaging the results for each track.

### Clustering of long-term cell tracks at shield region

Long-term cell tracks (after computing the SVF) were selected in a temporal interval from 4.5–7 hpf and in a spatial window around the shield region using the spherical coordinates of the cell positions and filtering for tracks with longitude *θ* in the range [−*π*/8, *π*/8]. All incomplete tracks (shorter than the desired time interval) were excluded from further analysis. For each track, the latitude φ was extracted for each time point and the respective profiles were clustered using *FindClusters* in Mathematica 11.3 with default parameters. We then selected the cluster corresponding to tracks that change latitudinal direction during the observation period for visualization. To visualize the major streamlines of this subset of tracks, we clustered the 3D spatial representation of tracks into 3 separate classes and computed the cluster centroids by averaging the spatial position of all tracks within a single cluster at each time point.

### Correlation of local single cell motion directions

For each cell at a particular time point we estimated the motion direction by taking the end-to-end vector of the local track segment (10 min time interval). We also computed the average motion direction for the six nearest neighbor cells in the same way. The vectors of the local motion direction of each cell and the average neighbor motion direction were split into its spherical coordinate components (r, *θ*, *ϕ*). At each time point, we computed the Spearman correlation over all pairs of cell and neighbor motion vectors separately for radius (r), longitude (*θ*, corresponding to D–V axis), and latitude (*ϕ*, corresponding to A-P axis).

### Quantifying the relative motion between layers

We computed the difference between the local motion vector for each mesendodermal cell (10 min intervals) and the averaged motion vector of its six nearest neighbors from the epiblast layer.

All differences were computed in spherical coordinates (*r*, *θ*, *ϕ*). The resulting difference vectors were mapped into 2D using the Mercator projection. The resulting irregular vector fields were averaged over a regular spatial grid for visualization. The radial component were visualized as smoothed histograms as this information is lost in the 2D maps.

### Density weighted streamlines

Flow fields representing directions of cell movement were modified using local cell density estimates. We first projected the cell positions to the unit sphere and mapped the resulting spherical coordinates into the Euclidean plane using the conformal (angle-preserving) Mercator projection. The trajectories were subdivided into 10 intervals in time, each spanning approximately 1.5 h. The spatial component was discretized using a regular 40 × 40 grid on the projections. For each single embryo *i*, we computed a vector field ${\boldsymbol{v}}_i^t\left( {\boldsymbol{x}} \right)$. The flow vector at each spatial grid cell represents the averaged normalized displacements extracted from all trajectories passing through this particular grid cell during the time interval *t*. The final flow field ***v***^*t*^(***x***) was obtained by averaging the flow at each grid cell over the samples. The width of each streamline was then scaled according to the sample count of the grid cell at its starting point. Additionally, we corrected for the area distortion in the Mercator projection, by dividing sample sizes by the area scaling factor sec^2^*ϕ* at the respective site x, with *ϕ* being the latitude at the central grid point.

### Correlation of cellular flows across layers

From the vector fields ${\boldsymbol{v}}_i^t({\boldsymbol{x}})$ of two different germlayers *i*, we calculated circular correlation^44^ using the following formula:

3 $$\begin{array}{*{20}{c}} {\rho \left( {\alpha ,\beta } \right) = \frac{{E\left( {\sin \left( {\alpha - \mu } \right)\sin \left( {\beta - \nu } \right)} \right)}}{{\sqrt {Var\left( {\sin \left( {\alpha - \mu } \right)} \right)Var\left( {\sin \left( {\beta - \nu } \right)} \right)} }},} \end{array}$$

with the circular observations *α*,*β* (flow directions from both germlayers at same position x in radians) and the respective central moments *μ* and *v*. Because *μ* and *v* are not unambiguously defined due to the uniform distribution of *α*,*β*, we set *μ* and *v* both to 0.

### Decomposition into movement components

The observed direction of movement ***v***^*t*^(***x***) at each sample point x was decomposed into the proportions associated with epiboly and convergence movement. As introduced in^25^ we first constructed reference vector fields representing pure epiboly **epi**(***x***) and convergence movement **conv**(***x***), and then locally projected the observed motion direction onto these reference directions: $s_{{\mathrm{epi}}}^t\left( {\boldsymbol{x}} \right)\,=\,{\boldsymbol{v}}^t\left( {\boldsymbol{x}} \right),{\mathbf{epi}}({\boldsymbol{x}})$and $s_{{\mathrm{conv}}}^t\left( x \right)\,=\,{\boldsymbol{v}}^t\left( {\boldsymbol{x}} \right),{\mathbf{conv}}(x)$, (Supplementary Fig. 6). The actual proportions for each movement component were then given by:

4 $$\begin{array}{*{20}{c}} {p^t\left( {\boldsymbol{x}} \right)\,=\,\left\{ {\begin{array}{*{20}{c}} {1 - 2\cos ^{ - 1}\left( {s^t\left( {\boldsymbol{x}} \right)} \right),if\;s^t\left( {\boldsymbol{x}} \right) \, > \, \, 0} \\ {0,\quad {\mathrm{otherwise}}} \end{array},} \right.} \end{array}$$

with $s^t\,=\,s_{{\mathrm{conv}}}^t$ or $s^t\,=\,s_{{\mathrm{epi}}}^t$. To summarize the fields *p*^*t*^(***x***) in a single value per time point, we calculated the mean across sites weighted by the number of observations *w*^*t*^(***x***) at ***x***:

5 $$\begin{array}{*{20}{c}} {o\left( t \right)\,=\,\frac{{\mathop {\sum }\nolimits_x p^t\left( {\boldsymbol{x}} \right)w^t\left( {\boldsymbol{x}} \right)}}{{\mathop {\sum }\nolimits_{\boldsymbol{x}} w^t\left( {\boldsymbol{x}} \right)}}.} \end{array}$$

### Edge-bundling of aggregated long-term cell tracks

To enable visual exploration of the large-scale tracking data we use an edge bundling method to simplify and declutter the visualization and highlight the main cell paths. We cluster all cell trajectories into spatially similar chunks^45^ and use a force-directed bundling^46^ algorithm to bring similar trajectories closer together. For rendering, we set a high level of transparency (95%) to highlight the main cell paths but also show the silhouettes of outliers.

### 3D rendering of cell positions

3D renderings of spherical cell centroids and tubular cell trajectories were created with Blender 2.77. Ambient occlusion was used to improve perception of depth ordering. The camera was set to orthographic to avoid perspective distortions.

### Tool for exploratory data analysis

A tool for the combined presentation of both the original and the bundled trajectory datasets was developed on the basis of the WebGL-library three.js. It can be executed in all common web browser, as well as on most mobile devices. The tool allows the user to explore the data, filter trajectories by properties or spatial position, map the trajectories into a 2.5D plane (mercator projection) and to learn about the dataset by following predefined tours (Supplementary Fig. 15, Supplementary Movie 7). The presented data contains all of the long-term trajectories, but for performance reasons the trajectories themselves are reduced to one fifth of their resolution. However, the data load is still relatively high (~60 MB) and thus requires an adequate internet connection. The visualization is rendered on the graphics card of the client computer. There are two different approaches on how to use the tool. First, it is possible to follow one of the guided tours. Select one of the tours (Supplementary Fig. 15) to start animations that will demonstrate highlights of the data. Second, the data can be explored highly interactive by mouse/touch input and a number of settings, which are explained in Supplementary Fig. 15. Furthermore, trajectories can be spatially filtered by pressing button s and using the mouse to select a region of interest. A double click resets the selection and shows all trajectories.

### Reporting summary

Further information on research design is available in the Nature Research Reporting Summary linked to this article.

## Supplementary information

Supplementary Information

Supplementary Video 1

Supplementary Video 2

Supplementary Video 3

Supplementary Video 4

Supplementary Video 5

Supplementary Video 6

Supplementary Video 7

Reporting Summary

Description of Additional Supplementary Files

## Data Availability

The authors declare that all data supporting the findings of this study are available within the article and its supplementary information files or from the corresponding author upon reasonable request. Image data for sample 1 (multi-channel, 3D time lapse after spatial registration and multi-view fusion) together with tracking results for each germlayer have been deposited in the Image Data Resource (https://idr.openmicroscopy.org) under accession code idr0068. More datasets generated during and/or analyzed during the current study are available from the corresponding authors on request. The long-term cell tracks can be interactively explored at https://imb-dev.gitlab.io/cell-flow-navigator/.

## Code availability

Source code (Wolfram Language/Mathematica) and notebook files (developed and tested with Mathematica 11.3 and 12.0) to reproduce cell movement analyses are available at https://gitlab.com/imb-dev/cell-flow-analysis/. A general, in-house tracking analysis package for Mathematica is available at https://github.com/kthr/Eidomatica and https://github.com/kthr/libEidomatica. Further Mathematica notebooks for data visualization are available upon request from N.S.
