# Supplementary material for: Multi-scale imaging and analysis identify pan-embryo cell dynamics of germlayer formation in zebrafish
Source: Nat Commun. 2019 Dec 17;10:5753. doi: 10.1038/s41467-019-13625-0 (PMC6917746; doi:10.1038/s41467-019-13625-0)
Supplement: Supplementary file 1 — Supplementary Information [file 41467_2019_13625_MOESM1_ESM.pdf]

Multi-scale imaging and analysis identifies pan-embryo cell dynamics of germlayer formation in zebrafish

- Supplementary Information –

Gopi Shah[1,2], Konstantin Thierbach[3,4], Benjamin Schmid[1,5], Johannes Waschke[4,6], Anna Reade[7,8], Mario Hlawitschka[6], Ingo Roeder[3], Nico Scherf[1,3,4] and Jan Huisken[1,9]

[1] Max Planck Institute of Molecular Cell Biology and Genetics, Pfotenhauerstr. 108, 01307 Dresden, Germany

[2] European Molecular Biology Laboratory, Carrer del Dr. Aiguader, 88, 08003 Barcelona, Spain

[3] Institute for Medical Informatics and Biometry, Carl Gustav Carus Faculty of Medicine, School of Medicine, TU Dresden, Fetscherstr. 74, 01307 Dresden, Germany

[4] Max Planck Institute for Human Cognitive and Brain Sciences, Stephanstr. 1a, 04103 Leipzig, Germany

[5] Optical Imaging Centre Erlangen, Friedrich-Alexander-University of Erlangen-Nuremberg, 91054 Erlangen, Germany

[6] Faculty of Computer Science and Media, Leipzig University of Applied Sciences, 04277 Leipzig, Germany

[7] Cardiovascular Research Institute, University of California, San Francisco, CA 94158-9001, USA

[8] Department of Biochemistry and Biophysics, University of California, San Francisco, CA 94158-2517, USA

[9] Morgridge Institute for Research, Madison, Wisconsin 53715, USA

Supplementary Figures 1- 15

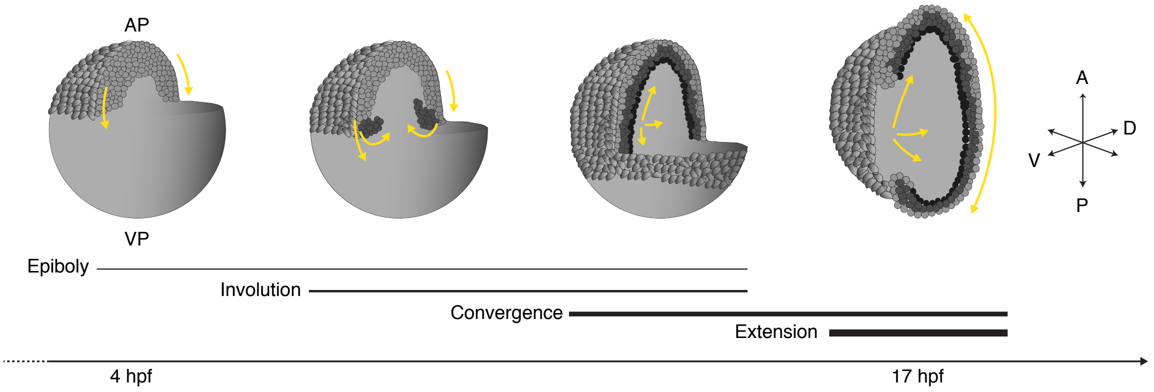

Supplementary Figure 1. Schematic of gastrulation movements.

Schematic showing the temporal overlap of the four major gastrulation movements - epiboly (movement from animal pole to vegetal pole), involution (inward movement), convergence (movement towards the dorsal midline) and extension (movement along the A-P axis).

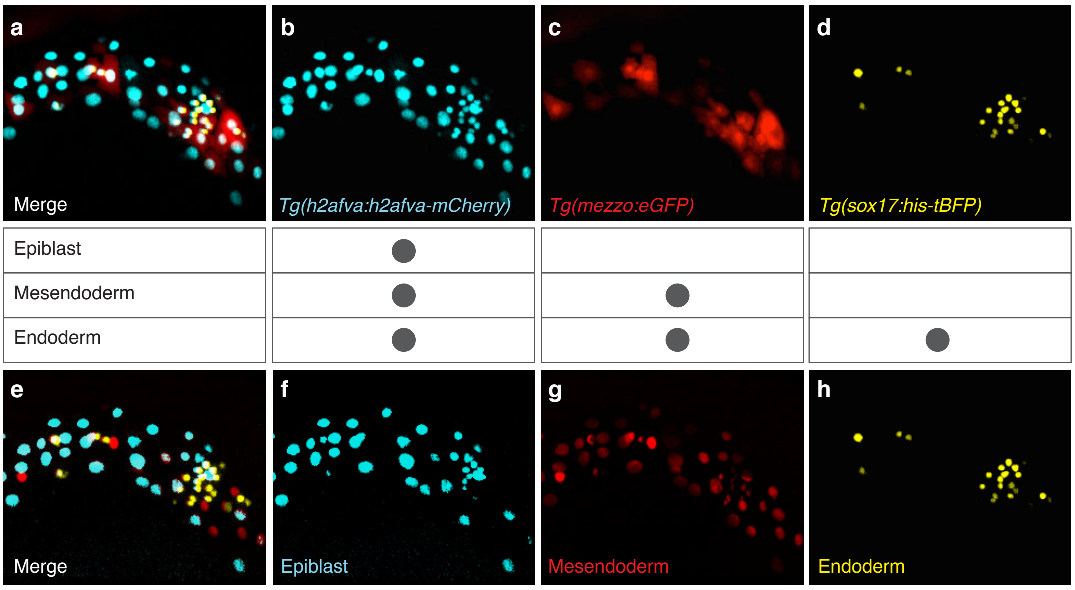

Supplementary Figure 2. Separation of signal from three transgenes into three germlayers.

(a) shows the merge of (b) Tg(h2afva:h2afva-mCherry) labeling nuclei of all cells, (c) cytoplasmic marker Tg(mezzo:eGFP), which labels mesendoderm cells and (d) Tg(sox17:H2B-tBFP) labelling the nuclei of endoderm cells only. (e-h) shows the separated nuclear signal of (e) all the three germlayers merged and the individual germlayers (f) epiblast (ectoderm), (g) mesoderm and (h) endoderm.

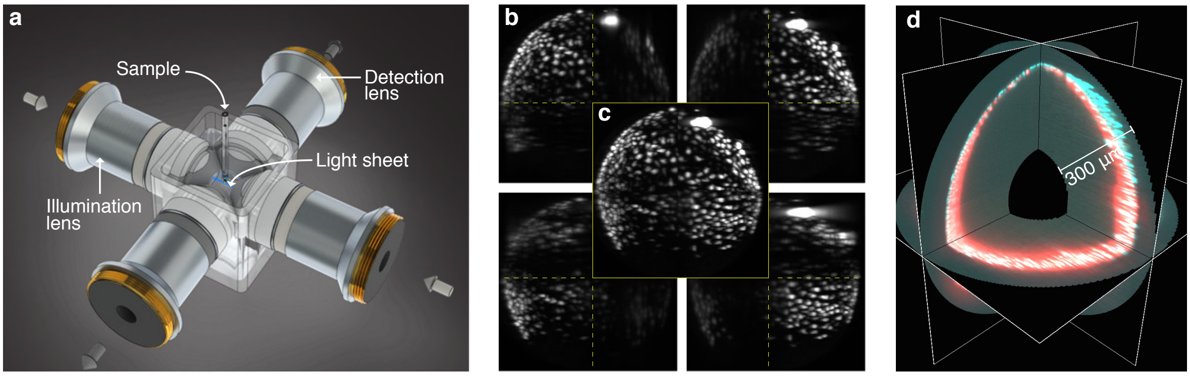

Supplementary Figure 3. Microscope design and image acquisition.

(a) The central unit of our 4-lens SPIM setup with two illumination and two detection lenses is shown. The sample is mounted from top, dipping into a water-filled sample chamber. (b) shows the four quadrants of raw data acquired by each combination of illumination and detection arm and a fusion of these images is shown in (c). (d) depicts the acquisition of masked raw data where the center of the embryo consisting of yolk and the blank space outside the embryo is masked to minimize the amount of data acquired. Cut out of the embryo is shown to make the masked black regions and the acquired 300 µm thick shell around the embryo surface visible.

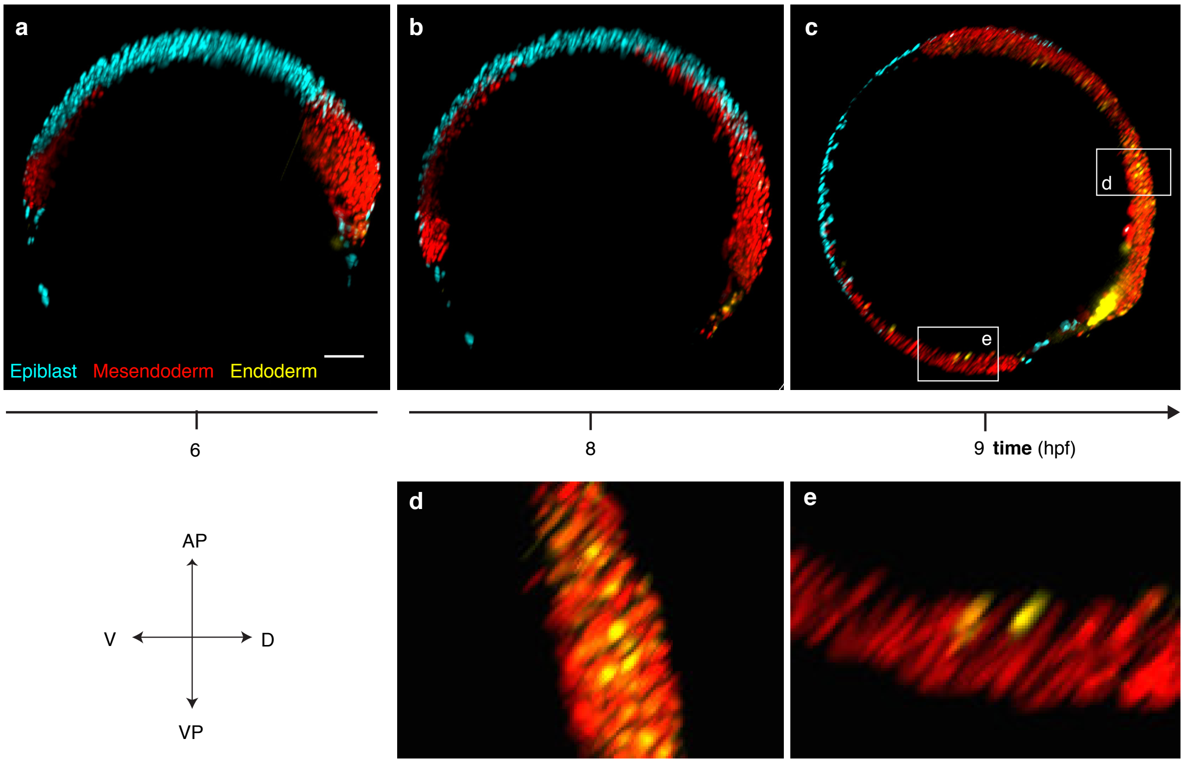

Supplementary Figure 4. Radial organization of germlayers.

(a-c) shows the stratification of germlayers. (a-b) Cells at the margin invaginate to form a two-layered embryo with outer epiblast (cyan) and inner mesendoderm (red). (c) Endoderm (yellow) cells stay mixed with the mesoderm cells (red) until the end of gastrulation and move to a deeper position thereafter (d, e), giving rise to a three-layered embryo. Scale bar: 100 μm.

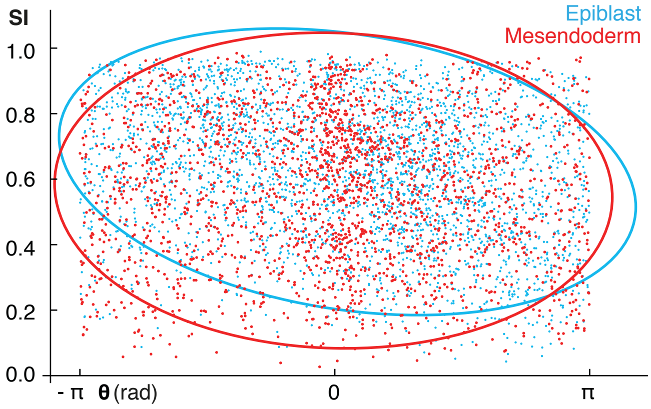

Supplementary Figure 5. Relationship between straightness of cell migration and longitudinal position.

Scatterplot of straightness index vs. longitude position θ (computed at the midpoint) for each trajectory (between 4.5 and 7 hpf of mesendoderm (red) and epiblast (blue) and the respective 90% prediction ellipsoids.

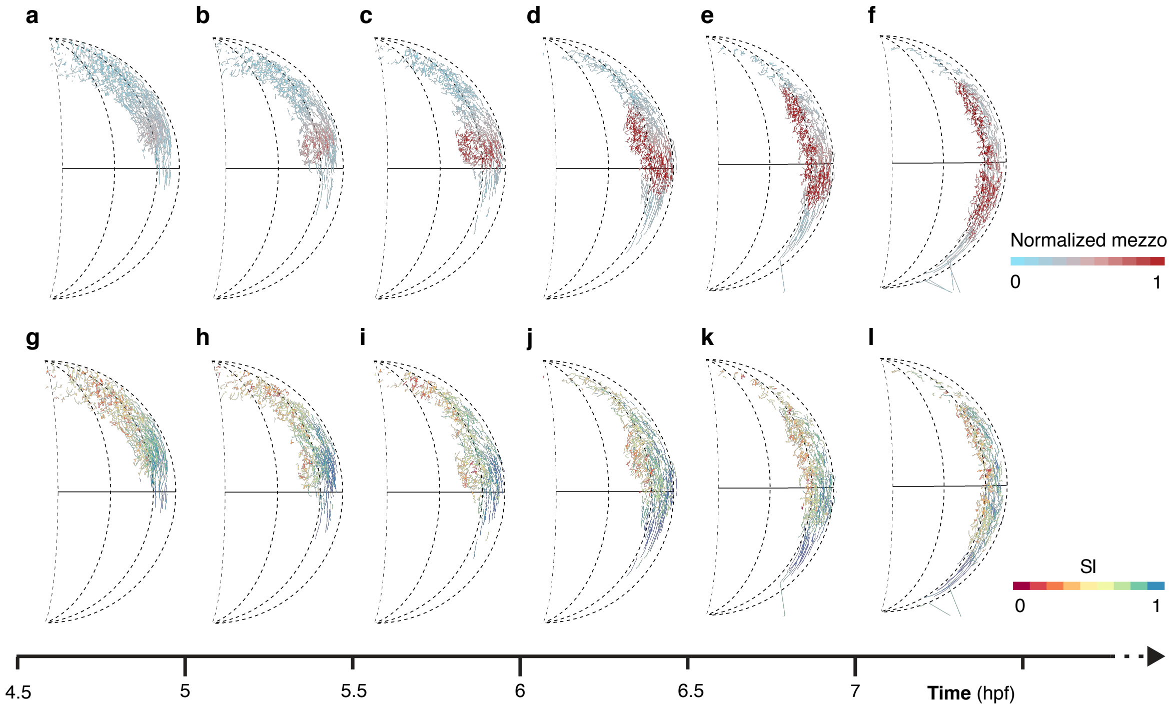

Supplementary Figure 6. Dynamics of internalization process around shield region.

(a-f) Lateral view of selected cell tracks in a spatial window around the shield region. Tracks are shown from 4.5 to 7.5 hpf in time intervals of 30 min. Color code indicates normalized *mezzo* expression along cell tracks. (g-l) Lateral view of same tracks as in (a-f) color-coded for straightness index (SI) of each track.

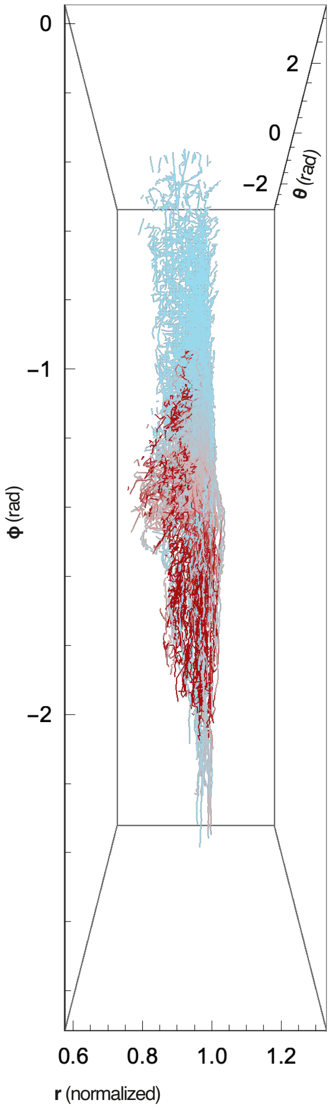

Supplementary Figure 7. Internalization at shield region in spherical coordinates.
Lateral view of selected cell tracks in a spatial window around the shield region visualized in spherical coordinate system. Tracks are shown from 4.5 to 7 hpf. Color code indicates normalized *mezzo* expression along cell tracks.

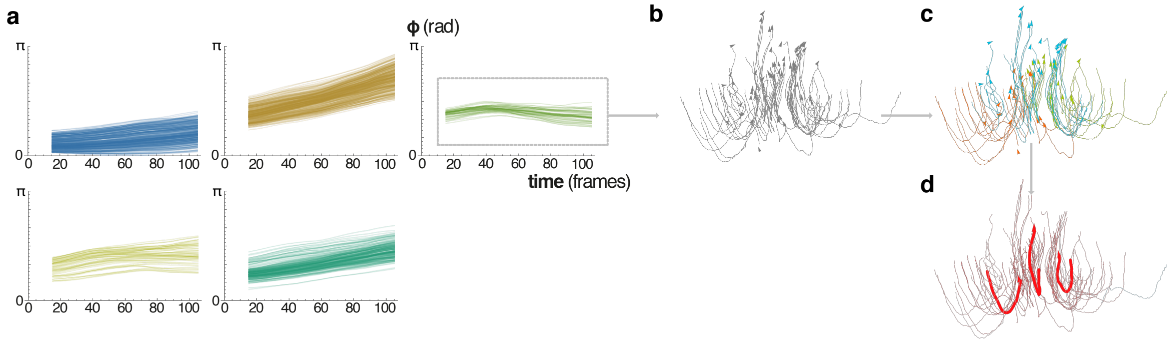

Supplementary Figure 8. Clustering of long-term tracks undergoing Internalization and AP movement.

(a) Line plots showing 1D profiles of latitude position across time for long-term tracks in the interval 4.5 - 7 hpf. The different clusters are shown as separate plots with random color code. Green cluster (highlighted by gray box) corresponds to cells undergoing internalization and movement towards animal pole. (b) 3D representation of tracks in the highlighted cluster. (c) Clustering of 3D tracks into three separate classes. (d) The three cluster centroids are shown as thick red lines over the 3D representation of tracks.

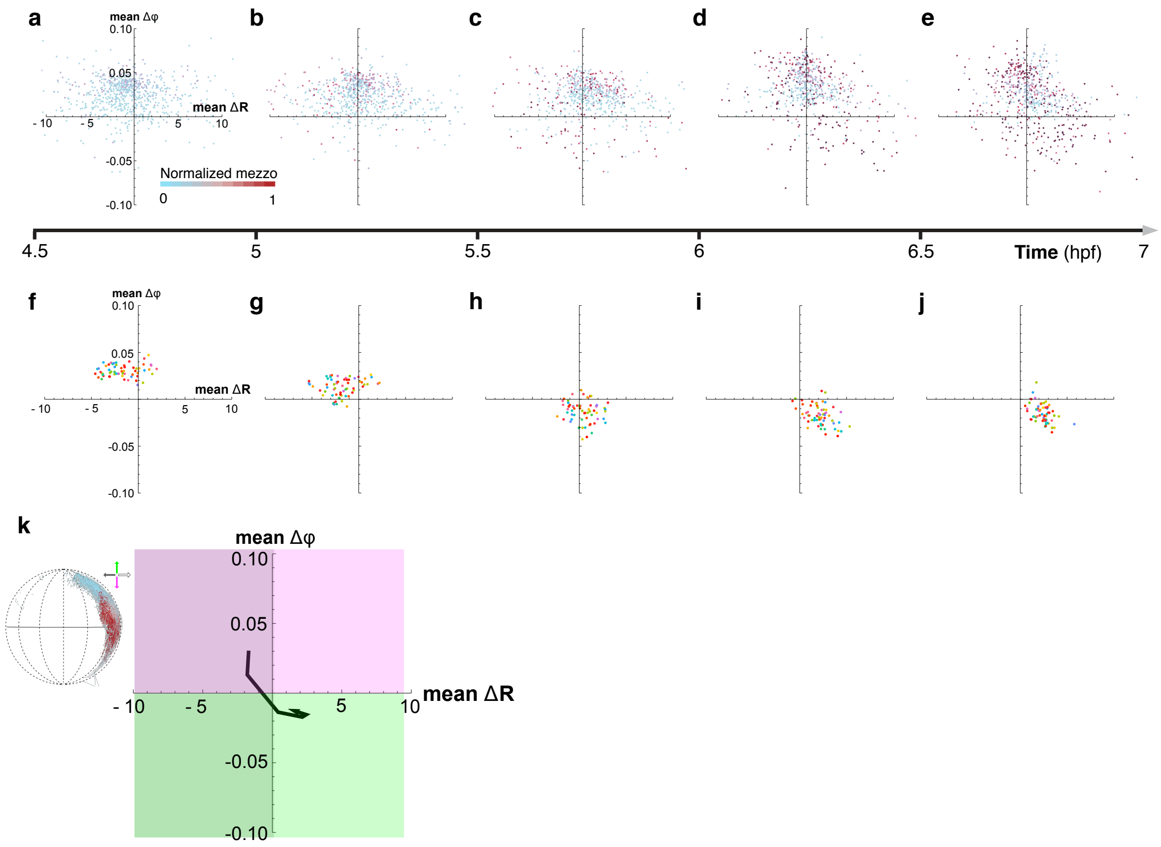

Supplementary Figure 9. Statistical analysis of internalization process around shield region.

(a-e) Scatterplots showing average change in radius (x axis) and average change in latitude (y axis) for each track in 30 min time intervals between 4.5 and 7 hpf. Color code indicates normalized *mezzo* expression for each track. (f-j) Scatterplots showing average change in radius (x axis) and average change in latitude (y axis) for clustered long-term cell tracks undergoing internalization and movement towards animal pole. Each individual track is assigned a random color. (k) Schematic plot highlighting the area of different movement directions for epiboly (magenta), animal pole (green), inward (dark), and outward (light) movement in the scatterplots (a-j). Thick arrow shows the average progression of changes in radius and latitude for the clustered cell tracks undergoing internalization and animal pole movement shown in (f-j) across 30 min time intervals from 4.5 to 7 hpf.

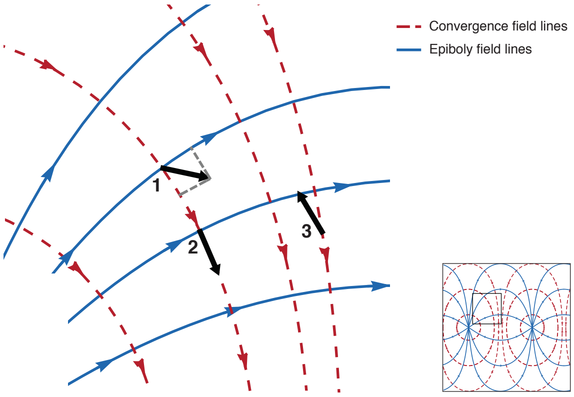

Supplementary Figure 10. Decomposition of flow directions into morphogenetic movement components.

Reference field lines for convergence (dashed-red) and epiboly (blue) of the region indicated by the box in the inset. The direction of the field lines is indicated by arrows in the respective color. Examples of potential flow directions are shown as black arrows. Dashed lines at arrow 1 indicate the projections onto the epiboly and convergence field lines. Vector 1 points in the direction of both epiboly and convergence, vector 2 solely points in epiboly direction and vector 3 has neither epiboly nor convergence component as it points in the opposite directions.

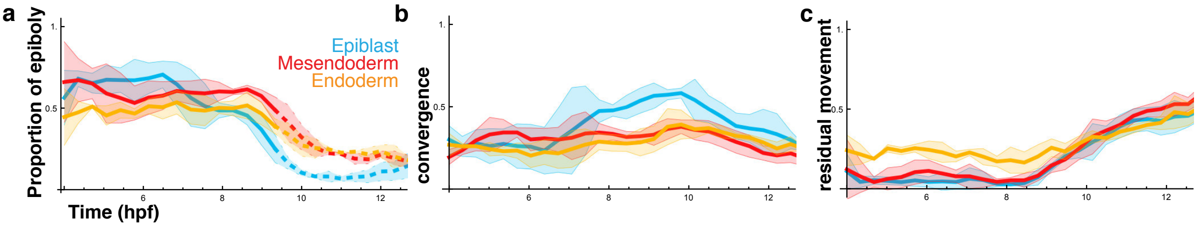

Supplementary Figure 11. Relative proportions of morphogenetic movements per germlayer.

Average proportions of epiboly (a) and convergence (b) and residual (c) movement for epiblast (blue), mesendoderm (red) and endoderm (yellow): Thick line shows mean (across n=3 embryos) and confidence bands indicate the region of mean +/- 1.96*standard error from 4 to 12.5 hpf. Dashed lines indicate the end of epiboly.

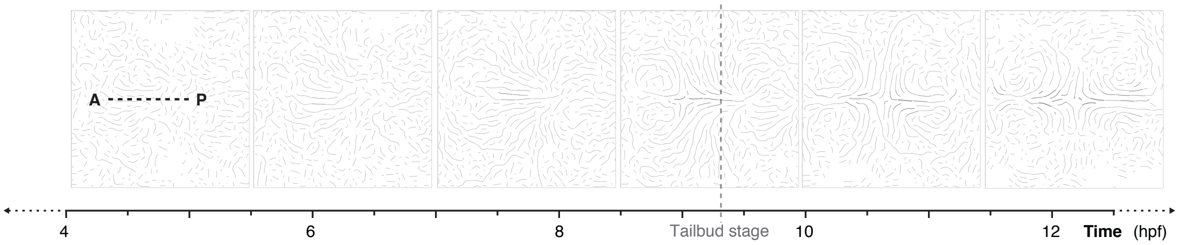

Supplementary Figure 12. Density weighted streamlines of endodermal layer.

Density weighted streamlines (see Fig. 4e) for endodermal cells. Thickness of streamlines indicates cell density at the respective site. Each interval covers about 1.5 hours of development.

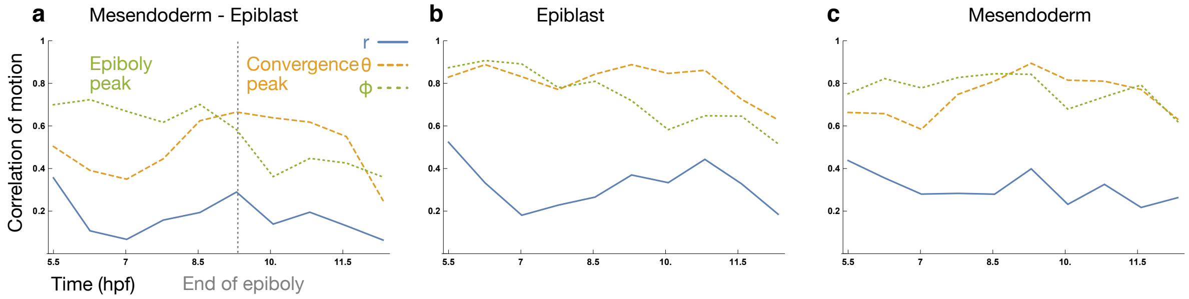

Supplementary Figure 13. Correlation of local cell motion direction. Plots showing the Spearman correlation at each time point between the local motion direction of each cell and the average motion direction of its six nearest neighbors. (a) Correlation between mesendoderm and epiblast cells. (b) Correlation within epiblast layer. (c) Correlation within mesendoderm layer. Motion directions were computed in spherical coordinates. Radial component (solid blue), longitude θ (corresponding to D-V motion) and latitude ϕ (corresponding to A-V motion) are shown separately.

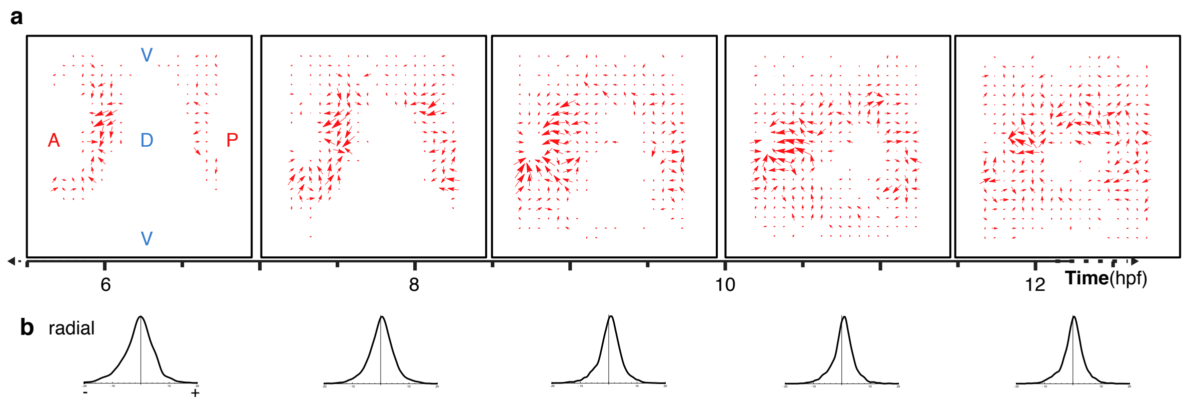

Supplementary Figure 14. Relative motion between mesoderm and ectoderm.

(a) Relative displacement from nearest ectodermal cells to each mesoderm cell, aggregated over spatial subregions for 90 min time-intervals shown on 2D Mercator projections. (b) Distribution of radial single-cell displacements (from nearest ectoderm to each mesoderm cell) across the same time intervals as (a).

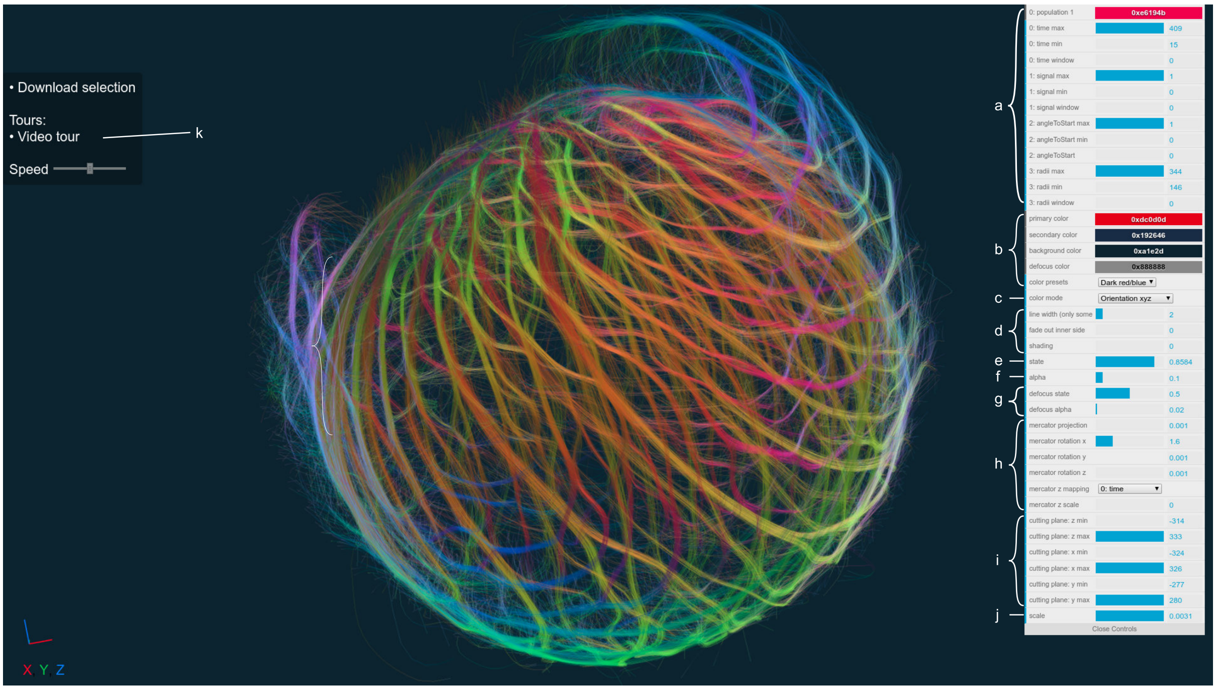

Supplementary Figure 15. Interactive data exploration.

Screenshot of the interactive data visualization running in a standard web browser. There are two different approaches on how to use explore the data: follow one of the guided tours or interactively interact with the data by mouse/touch input and a number of settings. The controls of the interactive visualization tool are:

(a) Filters for data attributes: time point; the measured signal; angle between a trajectory's local tangent and its initial tangent at time point 0; radius (distance to center). Only track segments matching these filters are rendered. If an attribute's window is set, the visualization only shows trajectories matching the attribute range from min to min + window (and max is ignored).

(b) The primary and secondary colors define the two ends of a color map that is used to visualize the attributes. In case the user has selected a region of interest, defocus color is used to stain unselected tracks. The tool also offers presets (dark and bright scheme).

(c) Various attributes (see (a)) and properties (orientation) can be color-mapped to the tracks.

(d) Further render options that improve spatial perception.

(e) Fade between original data and the bundled version.

(f) Transparency setting, which is especially useful for the presentation of the bundled tracks. High transparency hides outlying tracks but emphasizes dense regions.

(g) Like (e) and (f), but these values are only used for unselected tracks in case a region of interest has been defined.

(h) Mapping of the 3D tracks to a 2D plane via Mercator projection. Further parameters allow the rotation of the data (e.g. to place key features in the center of the 2D image) and to map data attributes (see (1)) as z coordinate.

(i) Cutting planes for all major axes.

(j) Scaling factor to let the data fit screen dimensions.

(k) Pre-defined tours to demonstrate features of the data.

Furthermore, trajectories can be spatially filtered by pressing button s and using the mouse to select a region of interest. A double click resets the selection and shows all trajectories.
