# Supplementary material for: Multi-scale imaging and analysis identify pan-embryo cell dynamics of germlayer formation in zebrafish
Source: Nat Commun. 2019 Dec 17;10:5753. doi: 10.1038/s41467-019-13625-0 (PMC6917746; doi:10.1038/s41467-019-13625-0)
Supplement: Supplementary file 9 — Reporting Summary [file 41467_2019_13625_MOESM9_ESM.pdf]

nature research

Corresponding author(s): Gopi Shah, Nico Scherf, Jan Huisken

Last updated by author(s): Oct 20, 2019

Reporting Summary

Nature Research wishes to improve the reproducibility of the work that we publish. This form provides structure for consistency and transparency in reporting. For further information on Nature Research policies, see Authors & Referees and the Editorial Policy Checklist.

Statistics

For all statistical analyses, confirm that the following items are present in the figure legend, table legend, main text, or Methods section.

n/a	Confirmed	
	X	The exact sample size (*n*) for each experimental group/condition, given as a discrete number and unit of measurement
X		A statement on whether measurements were taken from distinct samples or whether the same sample was measured repeatedly
X		The statistical test(s) used AND whether they are one- or two-sided<br>*Only common tests should be described solely by name; describe more complex techniques in the Methods section.*
X		A description of all covariates tested
	X	A description of any assumptions or corrections, such as tests of normality and adjustment for multiple comparisons
	X	A full description of the statistical parameters including central tendency (e.g. means) or other basic estimates (e.g. regression coefficient) AND variation (e.g. standard deviation) or associated estimates of uncertainty (e.g. confidence intervals)
X		For null hypothesis testing, the test statistic (e.g. *F*, *t*, *r*) with confidence intervals, effect sizes, degrees of freedom and *P* value noted<br>*Give P values as exact values whenever suitable.*
X		For Bayesian analysis, information on the choice of priors and Markov chain Monte Carlo settings
X		For hierarchical and complex designs, identification of the appropriate level for tests and full reporting of outcomes
X		Estimates of effect sizes (e.g. Cohen's *d*, Pearson's *r*), indicating how they were calculated

Our web collection on statistics for biologists contains articles on many of the points above.

Software and code

Policy information about availability of computer code

Data collection	Image acquisition was performed using a home-built microscope controlled with LabView. Data processing (Image registration, fusion, channel subtraction) was performed using Fiji, details of which are available in the methods section of the manuscript and upon request from authors.
Data analysis	Cell tracking was done using the TGMM software available at https://bitbucket.org/fernandoamat/tgmm-paper/src/master/<br>Data analysis was done using custom software written in Mathematica 11.3 and 12.0 (Wolfram Research, Inc.) available at https://gitlab.com/imb-dev/cell-flow-analysis or upon request from the authors.

For manuscripts utilizing custom algorithms or software that are central to the research but not yet described in published literature, software must be made available to editors/reviewers. We strongly encourage code deposition in a community repository (e.g. GitHub). See the Nature Research guidelines for submitting code & software for further information.

Data

Policy information about availability of data

All manuscripts must include a data availability statement. This statement should provide the following information, where applicable:

- Accession codes, unique identifiers, or web links for publicly available datasets
- A list of figures that have associated raw data
- A description of any restrictions on data availability

Data was deposited to the Image Data Resource (https://idr.openmicroscopy.org) under accession number idr0068. More datasets generated during and/or analysed during the current study are available from the corresponding authors on request. The long-term cell tracks can be interactively explored at https://imb-dev.gitlab.io/cell-flow-navigator/.

Field-specific reporting

Please select the one below that is the best fit for your research. If you are not sure, read the appropriate sections before making your selection.

[X] Life sciences [ ] Behavioural & social sciences [ ] Ecological, evolutionary & environmental sciences

For a reference copy of the document with all sections, see nature.com/documents/nr-reporting-summary-flat.pdf

Life sciences study design

All studies must disclose on these points even when the disclosure is negative.

Sample size	This is a study of wildtype samples only. Therefore, sample size was determined empirically based on the data points required for analysis. Each experiment in this manuscript requires 2-4 Terabytes of storage space and a large data processing infrastructure. With all these considerations and looking at the reproducibility of the datasets, we arrived at our optimal sample size values.
Data exclusions	No data were excluded from analysis
Replication	The samples for this study were blindly chosen from a pool of embryos laid by multiple zebrafish pairs. The experiment was repeated multiple times and most of the results shown are based on data collected from all samples.
Randomization	This study is performed on wildtype samples only and there was no allocation of samples into experimental groups.
Blinding	The samples for this study were blindly chosen from a pool of embryos laid by multiple zebrafish pairs. This study is performed on wildtype samples only, so there was no allocation of samples into experimental groups.

Reporting for specific materials, systems and methods

We require information from authors about some types of materials, experimental systems and methods used in many studies. Here, indicate whether each material, system or method listed is relevant to your study. If you are not sure if a list item applies to your research, read the appropriate section before selecting a response.

Materials & experimental systems

n/a	Involved in the study	
X		Antibodies
X		Eukaryotic cell lines
X		Palaeontology
	X	Animals and other organisms
X		Human research participants
X		Clinical data

Methods

n/a	Involved in the study	
X		ChIP-seq
X		Flow cytometry
X		MRI-based neuroimaging

Animals and other organisms

Policy information about studies involving animals; ARRIVE guidelines recommended for reporting animal research

Laboratory animals	Danio rerio [Fish lines: Tg(sox17:H2B-tBFP), Tg(mezzo:eGFP) and Tg(h2afva:h2afva-mCherry)]
Wild animals	n/a
Field-collected samples	n/a
Ethics oversight	Zebrafish were handled in accordance with EU directive 2011/63/EU as well as the German Animal Welfare Act.

Note that full information on the approval of the study protocol must also be provided in the manuscript.
