# Supplementary material for: Multi-scale imaging and analysis identify pan-embryo cell dynamics of germlayer formation in zebrafish
Source: Nat Commun. 2019 Dec 17;10:5753. doi: 10.1038/s41467-019-13625-0 (PMC6917746; doi:10.1038/s41467-019-13625-0)
Supplement: Supplementary file 10 — Description of Additional Supplementary Files [file 41467_2019_13625_MOESM10_ESM.pdf]

Title: Supplementary Movie 1:
Description: Gastrulation movements and specification of all three germlayers. Tg(h2afva:h2afva-mCherry) embryo labeling nuclei undergoing gastrulation movements from 4 to 18 hpf is shown (left). early - epiblast ; late - prospective ectoderm (cyan), mesendoderm (red) and endoderm (yellow) identity of the same embryo showing the formation and dynamics of all germlayers as they give rise to various organs (right).

Title: Supplementary Movie 2:
Description: Specification and dynamics of ectoderm, mesoderm and endoderm. Spatio-temporal dynamics during the formation of epiblast (cyan; early - epiblast ; late - prospective ectoderm), mesendoderm (red) and endoderm (yellow). Dynamics of these germlayers from 4 to 18 hpf leading to the formation of various organs is shown.

Title: Supplementary Movie 3:
Description: Registration of embryo to eliminate global movement. Images at two consecutive time points of the movie are aligned with each other to eliminate any global movement of the embryo itself and to preserve local cell movements. This movie compares the unregistered (left) and registered (right) data.

Title: Supplementary Movie 4:
Description: Cell tracking of all germlayers merged. Extracted cell trajectories between 4 and 14 hpf. Tracks of epiblast (cyan; early - epiblast ; late - prospective ectoderm), , mesendoderm (red) and endoderm (yellow). The current cell position is shown as a filled circle and the positions over the past 10 time points (corresponding to 20 min) as the trail of each track.

Title: Supplementary Movie 5:
Description: Radial and latitudinal cell organization at body axis. View of the zebrafish body-axis showing radial and latitudinal organization of germlayers. Cell centroids rendered as solid spheres, with cells belonging to early - epiblast ; late - prospective ectoderm (cyan), mesendoderm (red) and endoderm (yellow).

Title: Supplementary Movie 6:
Description: Cell tracking of all layers separated. Extracted cell trajectories between 4 and 14 hpf. Tracks of mesendoderm (red - middle), endoderm (yellow - right) and early - epiblast ; late - prospective ectoderm (cyan - left) are shown in separate views. Current cell position is shown as a filled circle and the positions over the past 10 time points (corresponding to 20 min) as the trail of each track.

Title: Supplementary Movie 7:
Description: Demo of interactive, exploratory data visualization. Different mappings of long-term cell tracks showing exploratory data analysis with the interactive data viewer. The tour demonstrates the different visualizations shown in Fig. 4.
